# Exclusive Characteristics of the p.E555K Dominant-Negative Variant in Autosomal Dominant E47 Deficiency

**DOI:** 10.1007/s10875-024-01758-x

**Published:** 2024-07-29

**Authors:** Takanori Utsumi, Miyuki Tsumura, Masato Yashiro, Zenichiro Kato, Kosuke Noma, Fumiaki Sakura, Reiko Kagawa, Yoko Mizoguchi, Shuhei Karakawa, Hidenori Ohnishi, Charlotte Cunningham-Rundles, Peter D. Arkwright, Masao Kobayashi, Hirokazu Kanegane, Dusan Bogunovic, Bertrand Boisson, Jean-Laurent Casanova, Takaki Asano, Satoshi Okada

**Affiliations:** 1 Department of Pediatrics, Graduate School of Biomedical and Health Sciences, Hiroshima University, Hiroshima, Japan; 2 Department of Pediatrics, Okayama University Hospital, Okayama, Japan; 3 Department of Pediatrics, Graduate School of Medicine, Gifu University, Gifu, Japan; 4 Structural Medicine, United Graduate School of Drug Discovery and Medical Information Science, Gifu University, Gifu, Japan; 5 Department of Applied Genomics, Kazusa DNA Research Institute, Chiba, Japan; 6 Division of Allergy and Clinical Immunology, Departments of Medicine and Pediatrics, Icahn School of Medicine at Mount Sinai, New York, NY USA; 7 Lydia Becker Institute of Immunology and Inflammation, University of Manchester, Manchester, UK; 8 Japanese Red Cross Chugoku-Shikoku Block Blood Center, Hiroshima, Japan; 9 Department of Child Health and Development, Graduate School of Medical and Dental Sciences, Tokyo Medical and Dental University (TMDU), Tokyo, Japan; 10 Center for Inborn Errors of Immunity, Icahn School of Medicine at Mount Sinai, New York, NY USA; 11 Precision Immunology Institute, Icahn School of Medicine at Mount Sinai, New York, NY USA; 12 Mindich Child Health and Development Institute, Icahn School of Medicine at Mount Sinai, New York, NY USA; 13 Department of Pediatrics, Icahn School of Medicine at Mount Sinai, New York, NY USA; 14 Department of Microbiology, Icahn School of Medicine at Mount Sinai, New York, NY USA; 15 St. Giles Laboratory of Human Genetics of Infectious Diseases, Rockefeller Branch, The Rockefeller University, New York, NY USA; 16 Laboratory of Human Genetics of Infectious Diseases, Necker Branch, INSERM U1163, Necker Hospital for Sick Children, Paris, France; 17 Paris Descartes University, Imagine Institute, Paris, France; 18 Pediatric Hematology-Immunology Unit, Necker Hospital for Sick Children, AP-HP, Paris, France; 19 Howard Hughes Medical Institute (HHMI), New York, NY USA; 20 Department of Genetics and Cell Biology, Research Institute for Radiation Biology and Medicine, Hiroshima University, Hiroshima, Japan

**Keywords:** TCF3, E47, E555K, Dominant-negative, B-cell deficiency, Agammaglobulinemia

## Abstract

**Purpose:**

*Transcription factor 3* (*TCF3*) encodes 2 transcription factors generated by alternative splicing, E12 and E47, which contribute to early lymphocyte differentiation. In humans, autosomal dominant (AD) E47 transcription factor deficiency is an inborn error of immunity characterized by B-cell deficiency and agammaglobulinemia. Only the recurrent de novo p.E555K pathogenic variant has been associated with this disease and acts via a dominant-negative (DN) mechanism. In this study, we describe the first Asian patient with agammaglobulinemia caused by the *TCF3* p.E555K variant and provide insights into the structure and function of this variant.

**Methods:**

*TCF3* variant was identified by inborn errors of immunity-related gene panel sequencing. The variant E555K was characterized by alanine scanning of the E47 basic region and comprehensive mutational analysis focused on position 555.

**Results:**

The patient was a 25-year-old male with B-cell deficiency, agammaglobulinemia, and mild facial dysmorphic features. We confirmed the diagnosis of AD E47 transcription factor deficiency by identifying a heterozygous missense variant, c.1663 G>A; p.E555K, in *TCF3*. Alanine scanning of the E47 basic region revealed the structural importance of position 555. Comprehensive mutational analysis focused on position 555 showed that only the glutamate-to-lysine substitution had a strong DN effect. 3D modeling demonstrated that this variant not only abolished hydrogen bonds involved in protein‒DNA interactions, but also inverted the charge on the surface of the E47 protein.

**Conclusions:**

Our study reveals the causative mutation hotspot in the *TCF3* DN variant and highlights the weak negative selection associated with the *TCF3* gene.

**Supplementary Information:**

The online version contains supplementary material available at 10.1007/s10875-024-01758-x.

## Introduction

*Transcription factor 3* (*TCF3*) encodes 2 basic helix-loop-helix (bHLH) transcription factors, E12 and E47 [1, 2]. These proteins are members of the E-protein family and activate transcription by binding to palindromic DNA sequences (-CANNTG-), called E-box [3, 4]. They can form homodimers or heterodimers with other tissue-specific bHLH proteins [1, 2, 5–7]. They play crucial roles in the early differentiation of various organs, including hematopoietic organs, muscles, and nerves [8, 9]. In bone marrow hematopoietic cells, E47 mainly forms a homodimer and induces downstream B lineage-specific gene expression [9–12]. E47 expression is not only essential for the commitment of hematopoietic stem cells to the B-cell lineage, but also important for the maintenance of B-cell development in the bone marrow [9, 13–17].

E47 transcription factor deficiency results in early arrest of B-cell differentiation, leading to B-cell deficiency and agammaglobulinemia [18–21]. Recently, 2 other allelic forms of TCF3 deficiency have been described: autosomal dominant (AD) and autosomal recessive (AR) [18–26]. The latter impacts both TCF3 isoforms E12 and E47. Complete AR TCF3 deficiency is associated with reduced B cells, hypogammaglobulinemia, dysmorphic phenotypes, and B-cell acute lymphoblastic leukemia (B-ALL) [21, 22]. AD TCF3 deficiency can be divided into 2 additional categories. (i) TCF3 haploinsufficiency (HI) also impacts both TCF3 isoforms, resulting in common variable immunodeficiency (CVID) with low B cells and hypogammaglobulinemia [21, 25]. In TCF3 HI, the penetrance of the immunological phenotype is almost complete, whereas the clinical phenotype is incomplete. (ii) E47 transcription factor deficiency, which is the original AD TCF3 deficiency, impacts only E47 through a dominant-negative (DN) mechanism [18–21]. The *TCF3* DN variant leading to qualitative impairment of the E47 transcription factor was first described in 2013 in 4 independent patients with recurrent bacterial infections and agammaglobulinemia [18, 19]. These unrelated patients shared the common variant *TCF3* E47-p.E555K [19]. Subsequently, 2 additional patients with the same variant were reported by different institutions [20, 21]. Boisson et al. [19] reported that the E555K variant has a DN effect, dimerizing with wild-type (WT) E47 and depriving it of its DNA-binding ability. However, the molecular and functional understanding associated with the exclusivity of p.E555K as a disease-causing variant in *TCF3* DN remains incomplete.

In this study, we report the first Asian patient with the *TCF3* p.E555K DN variant and elucidate the unique features that contribute to its exclusivity.

## Materials and Methods

### Genetic Analysis

Genetic analysis was performed after obtaining written informed consent from the patient and his parents. We extracted genomic DNA from their whole blood. We then subjected the samples to inborn errors of immunity (IEI)-related gene panel sequencing based on the 2017 update of the classification by the expert committee of the International Union of Immunological Societies [27, 28]. We confirmed the identified variant by Sanger sequencing.

### Protein Structure Modeling and Analysis

Due to the lack of available structural data on the E47 homodimer, the human SCL: E47 heterodimer (PDB ID: 2YPB) was used as a template for the 3D structural model of the E47 homodimer binding to the E-box (https://www.rcsb.org/) [29]. The model of the E555K variant was generated using Molecular Operating Environment (MOE) 2013.08 (Chemical Computing Group, Inc., Montreal, Canada, 2013; https://www.chemcomp.com/). All structural data were visualized with PyMOL software (https://pymol.org/2/). The electrostatic potential at the protein surface was calculated using the Adaptive Poisson-Boltzmann Solver (APBS) plug-in for PyMOL.

### Expression Vectors

The pCMV6 mammalian expression vector encoding WT E47 was obtained from Rockefeller University [19]. Using site-directed mutagenesis techniques, all amino acids from R547 to V559 encoded by the vector, except for 2 alanine residues (A550 and A553), were individually substituted with alanine for alanine scanning. Similarly, the glutamate at position 555 was replaced with all other 19 amino acids and a stop codon (*) for comprehensive mutational analysis.

### Immunoblot Analysis

Human embryonic kidney (HEK) 293T cells were plated at 1.25 × 10^5^ cells/well in 24-well plates and cultured for 16 h at 37 °C in the presence of 5% CO_2_ in DMEM (Thermo Fisher Scientific, Waltham, MA, USA) containing 10% heat-inactivated fetal bovine serum supplemented with 100 µg/mL penicillin/streptomycin. A vector carrying the E47 WT allele or each E47 variant allele was then transfected into HEK293T cells using Lipofectamine LTX Reagent (Thermo Fisher Scientific) according to the manufacturer’s protocol. After 24 h, the transfected HEK293T cells were lysed in RIPA lysis buffer (Sigma‒Aldrich, St. Louis, MO, USA) supplemented with protease and phosphatase inhibitor cocktails (Thermo Fisher Scientific). Whole-cell protein extracts were separated by SDS‒PAGE and transferred to polyvinylidene fluoride membranes (Merck KGaA, Darmstadt, Germany). The membranes were blocked with 10% skim milk (Becton Dickinson, Franklin Lakes, NJ, USA) for 60 min at room temperature and incubated overnight at 4 °C with mouse anti-human E47 antibody (1:2,000 dilution; RRID: AB_395228, BD Biosciences, Franklin Lakes, NJ, USA) and anti-GAPDH antibody (1:1,000 dilution; RRID: AB_1078991, Sigma‒Aldrich) as primary antibodies. Horseradish peroxidase (HRP)-conjugated anti-mouse antibody (1:2,000 dilution; RRID: AB_772210, Cytiva, Malborough, MA, USA) was used as secondary antibody. Antibody binding was detected by chemiluminescence using an ImmunoStar Zeta (Fujifilm Wako Pure Chemical Corporation, Osaka, Japan). Three independent experiments were performed to confirm the results.

### Luciferase Reporter Assay

Luciferase reporter assay was performed as previously described [19]. HEK293T cells were plated at a density of 3.0 × 10^4^ cells/well in 96-well plates and cultured for 16 h. The indicated doses of E47 WT and/or each E47 variant vector (adding the empty vector up to a total dose of 100 ng) were co-transfected into HEK293T cells with the pGL4 firefly luciferase reporter vector and the *Renilla* luciferase reporter vector (pRL-TK; Promega, Madison, WI, USA) in the presence of Lipofectamine LTX Reagent. The firefly luciferase reporter vector contains 2 copies of the µE5-µE2 sequence, which are E47 binding sites: µE5 (-CACCTG-) and µE2 (-CAGCTG-). After 24 h, E47 transcriptional activity was assessed using the Dual-Glo Luciferase Assay System (Promega) according to the manufacturer’s protocol. E47 transcriptional activity was calculated from the ratio of firefly/*Renilla* luciferase luminescence. The values obtained were normalized to the value of WT 3.0 ng, which corresponds to 100%. Each experiment was performed 3 times independently and indicated as the mean ± SEM. The E47 transcriptional activity of WT 1.5 ng was considered to reflect a monoallelic state, and that of WT 3.0 ng was considered to reflect a biallelic (normal) state. Negative dominance was defined based on (i) transcriptional activity below that of WT 1.5 ng when co-transfected with the same amount (1.5 ng) of each variant and (ii) dose dependence.

### Subcellular Protein Fractionation and Co-Immunoprecipitation

HEK293T cells were plated at a density of 6.25 × 10^5^ cells/well in 6-well plates and cultured for 16 h. The V5-tagged E47 WT vector and the FLAG-tagged E47 WT or each variant vector were co-transfected into HEK293T cells using Lipofectamine LTX Reagent. After 24 h, the transfected HEK293T cells were separated into cytoplasmic and nuclear extract samples according to the manufacturer’s protocol using the NE-PER Nuclear and Cytoplasmic Extraction Reagents (Thermo Fisher Scientific). Co-immunoprecipitation samples were prepared using the Pierce Co-immunoprecipitation Kit (Thermo Fisher Scientific) with rabbit anti-V5 antibody (RRID: AB_261889, Sigma‒Aldrich) or negative control normal rabbit IgG antibody (RRID: AB_145841, Merck KGaA). Proteins in each sample were separated by SDS‒PAGE and detected by immunoblotting with primary antibodies: rabbit anti-V5 (1:2,000 dilution), mouse anti-FLAG (1:2,000 dilution; RRID: AB_259529, Sigma–Aldrich), rabbit anti-lamin A/C (1:500 dilution; RRID: AB_648154, Santa Cruz Biotechnology, Dallas, TX, USA), and mouse anti-GAPDH (1:1,000). HRP-conjugated anti-mouse (1:2,000) and anti-rabbit (1:2,000 dilution; RRID: AB_2722659, Cytiva) antibodies were used as secondary antibodies, and Clean-Blot IP Detection Reagent (Thermo Fisher Scientific) was used for co-immunoprecipitation. Three independent experiments were performed to validate the results.

## Results

### Clinical and Genetic Features

**Fig. 1:**
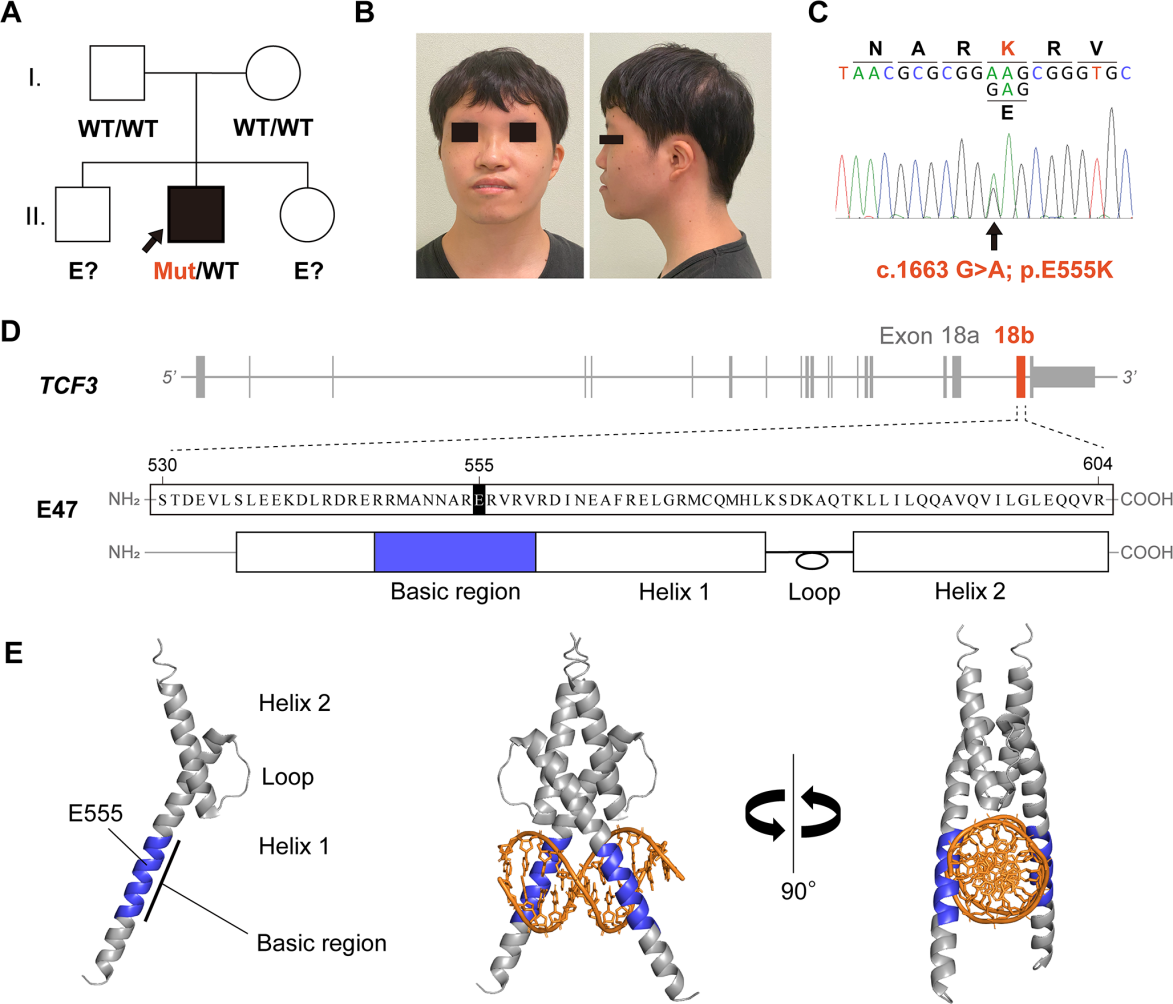
Identification of the *TCF3*DN variant. (**A**) Family pedigree of the patient. The arrow indicates the affected patient. *WT*, wild-type; *Mut*, mutant; *E?*, unknown genotype. (**B**) Facial features of the patient. (**C**) Sanger sequencing shows the heterozygous single base substitution c.1663 G>A; p.E555K (E47) in *TCF3*. (**D**) Schematic diagram of the *TCF3* gene (top), and the primary structure (middle) and secondary structure (bottom) of the E47 protein (residues S530–R604). Exon 18b, which is specific to the E47 isoform, consists of 75 amino acids and contains a basic helix-loop-helix (bHLH) domain. (**E**) 3D structural model of the E47 monomer (left) and the homodimer bound to the E-box sequence (center and right). The area highlighted in blue represents the basic region

The patient was a 25-year-old Japanese man born to non-consanguineous parents. His parents, older brother, and younger sister were all healthy and had no history of immunodeficiency (Fig. 1A). Despite being small for gestational age (39 weeks’ gestation, birth weight of 2,434 g; 2.7th percentile on the Japanese growth chart) and experiencing mild asphyxia with meconium-stained amniotic fluid at birth, he developed well during the first months of life. He received the oral polio vaccine at 4 months of age without sequelae. At 6 months of age, he developed acute bacterial pneumonia and agammaglobulinemia (IgG 0.09 g/L, IgA 0.01 g/L, IgM 0.01 g/L). He was treated with intravenous immunoglobulin (IVIG) and antibiotics. Subsequently, regular IVIG therapy was initiated to prevent serious infections due to agammaglobulinemia. After 8 years without a serious infection, immunological evaluation of peripheral blood by immunophenotyping revealed a mildly reduced number of peripheral CD20^+^ B cells (3.9%), but normal numbers of CD3^+^ T cells and CD16^+^CD56^+^ natural killer (NK) cells. Although antimicrobial prophylaxis was initiated, his infections could not be completely prevented. He suffered from pneumonia caused by *Haemophilus influenzae* every few years. At 22 years of age, IVIG was switched to subcutaneous immunoglobulin therapy with serum IgG trough levels maintained at approximately 10 g/L. Since the change in treatment, the frequency and severity of his infections have decreased.

In addition to the immunological phenotypes, he presented with non-immune manifestations characterized by mild facial dysmorphic features, a webbed neck, single palmar creases, and growth failure with growth hormone deficiency since childhood. Facial features included low-set ears, hypertelorism, downslanted palpebral fissures, a saddle nose, and a short philtrum with an upturned upper lip (Fig. 1B). Although Down syndrome was initially suspected, his karyotype showed a typical male pattern, 46,XY. His short stature (111.8 cm, − 3.27 SD) was treated with recombinant human GH starting at 8 years of age, but the response was suboptimal (his final height was 151.7 cm, − 3.28 SD).

At the age of 25, he visited our hospital for an immunological and genetic reassessment. Deep immunophenotyping of peripheral blood mononuclear cells (PBMCs) by multicolor flow cytometry (Supplementary Methods) [30] revealed a complete B-cell deficiency. CD19^+^ and CD20^+^ B cells were undetectable, while the subsets of CD3^+^ T cells and CD16^+^CD56^+^ NK cells remained nearly normal (Table S1, Fig. S1). Genetic analysis using an IEI-related gene panel sequencing identified a heterozygous missense variant, c.1663 G > A; p.E555K, in the *TCF3* gene (Fig. 1C). No significant variants were identified in other IEI-related genes. Subsequent genetic analysis of his parents revealed that the identified variant was de novo. The expression of the E47 proteins in the patient’s PBMCs was comparable to that of the healthy control (Fig. S2).

The *TCF3* gene encodes the transcription factors E12 and E47, which are generated by alternative splicing of exon 18 (18a and 18b) (Fig. 1D). These exons contain the bHLH domain, a crucial region for these proteins to function as transcription factors (Fig. 1D). In hematopoietic cells, the translated E47 proteins form homodimers via their helix-loop-helix domains and bind specifically to the E-box sequence via 2 basic regions (Fig. 1E). The identified p.E555K variant was located within the basic region of the E47-specific exon 18b (Fig. 1D, E).

### Literature Review of TCF3 Deficiency

We summarized the literature on TCF3 deficiency to highlight the characteristics of AD E47 transcription factor deficiency (Table 1). A total of 30 individuals were described in 8 reports [18–25]. AR and AD TCF3 deficiencies were included in 8 and 22 individuals, respectively. Patients with AD TCF3 deficiency consisted of 7 clinically symptomatic patients and 9 carriers with *TCF3* HI variants, besides 6 patients with the *TCF3* DN variant. AR TCF3 deficiency was found in 8 patients and was caused by homozygous loss-of-function (LOF) variants in *TCF3*. This variant resulted in a complete loss of TCF3 protein, affecting both E12 and E47. Patients with AR TCF3 deficiency exhibited relatively broad phenotypes, including not only B-cell deficiency and agammaglobulinemia, but also facial dysmorphic features and B-ALL [21–24]. The recently defined TCF3 HI is caused by heterozygous LOF variants that reduce WT TCF3 expression without complete absence, presenting milder phenotypes such as CVID or even asymptomatic with incomplete penetrance [21, 22, 24, 25]. TCF3 HI presented with hypogammaglobulinemia, reduced total B cells, switched memory B cells, plasmablasts, and an intermediate reduction of in vitro plasmablast generation and immunoglobulin production [21, 25]. A patient with a *TCF3* HI variant and a heterozygous *TNFRSF13B* variant was diagnosed with severe CVID and systemic lupus erythematosus affected by epistasis [25]. In contrast, the heterozygous *TCF3* DN variant was highly deleterious despite unimpaired protein expression, resulting in complete B-cell deficiency and agammaglobulinemia [18–21]. Intriguingly, the only *TCF3* DN variant identified to date is p.E555K [19–21].

**Table 1:** Clinical and genetic summary of patients with TCF3 deficiency

Pt.	Inheritance	Variant	Zygosity	Mechanism	Ethnicity	Sex	Age	Onset	Infections	Other findings	CD19^+^B cells (/µL) / (% of lymphocytes)	Hypo/Agammaglobulinemia	IgG (g/L)	IgA (g/L)	IgM (g/L)	Ref.
1	AR	c.808 C>T p.Q270*	Homo	LOF	Tunisian	M	10 years	Early childhood	Recurrent pneumonia and meningitis	Facial dysmorphic features, B-ALL	NA / 0.1	Yes	NA	NA	NA	[22]
2	AR	c.808 C>T p.Q270*	Homo	LOF	Tunisian	F	13 years	Early childhood	Recurrent pneumonia	Facial dysmorphic features, failure to thrive	NA / 3.5	Yes	6.75	0.67	0.18	[22]
3	AR	Exons 5–11 deletion	Homo	LOF	NA	F	9 years	1 year	Recurrent pneumonia, chronic diarrhea	Anemia, failure to thrive	49 / 1.2	Yes	1.11	< 0.15	0.14	[23]
4	AR	c.380 C>G p.S127*	Homo	LOF	Iranian	F	32 years	7 years	Recurrent otitis, sinusitis, pneumonia, herpetic encephalitis	Mild hearing loss due to herpetic encephalitis	0 / 0.2	Yes	4.30	0.07	0.22	[24]
5	AR	c.1451–18 A>T p.G486Lfs*4	Homo	LOF	NA	M	6 years	NA	Recurrent pneumonia	Facial dysmorphic features, B-ALL	0 / 0	Yes	1.37	0.07	0.32	[21]
6	AR	c.1451–18 A>T p.G486Lfs*4	Homo	LOF	NA	M	3 years	NA	No	Facial dysmorphic features	116 / 2.5	Yes	2.95	0.12	0.11	[21]
7	AR	c.1451–18 A>T p.G486Lfs*4	Homo	LOF	NA	F	8 months	NA	No	Facial dysmorphic features, B-ALL	210 / 3.0	Yes	2.30	0.31	0.12	[21]
8	AR	c.1326 + 5 G>A p.L442_V443ins27	Homo	LOF	NA	F	7 years	NA	Bacteremic pneumococcal pneumonia, EBV-associated leukocytosis with lymphocytosis	Facial dysmorphic features, mild intellectual disability	396 / 8.2	Yes	7.52^a^	0.27	0.17	[21]
9	AD	c.808 C>T p.Q270*	Hetero	HI	Tunisian	M	49 years	No	No	No	NA / 1.9	No	8.91	3.21	0.21	[22]
10	AD	c.808 C>T p.Q270*	Hetero	HI	Tunisian	F	49 years	No	No	No	NA / 2.0	No	10.23	1.13	0.59	[22]
11	AD	c.808 C>T p.Q270*	Hetero	HI	Tunisian	M	10 years	No	No	No	NA / 2.4	No	9.25	1.51	0.37	[22]
12	AD	c.380 C>G p.S127*	Hetero	HI	Iranian	M	NA	No	No	No	NA	NA	NA	NA	NA	[24]
13	AD	c.380 C>G p.S127*	Hetero	HI	Iranian	F	NA	No	No	No	NA	NA	NA	NA	NA	[24]
14	AD	c.380 C>G p.S127*	Hetero	HI	Iranian	M	NA	No	No	No	NA	NA	NA	NA	NA	[24]
15	AD	c.1451–18 A>T p.G486Lfs*4	Hetero	HI	NA	M	26 years	No	No	No	175 / 5.3	No	12.62	3.12	0.45	[21]
16	AD	c.1451–18 A>T p.G486Lfs*4	Hetero	HI	NA	F	23 years	No	No	No	50 / 7.0	Yes	7.26	0.39	0.35	[21]
17	AD	c.1541 C>A p.S514*	Hetero	HI	NA	M	10 years	NA	Varicella, impetigo, oral thrush, gastroenteritis	No	136 / 4.1	Yes	9.01^a^	0.28	0.18	[21]
18	AD	c.599_600delinsGCT p.Y200Cfs*54	Hetero	HI	NA	F	53 years	NA	Recurrent and chronic sinus infection	Asthma, granulomatous lung disease, obesity, type 2 diabetes, diabetes insipidus	124 / 8.7	Yes	4.86	1.62	0.10	[21]
19	AD	c.1326 + 5 G>A p.V433*	Hetero	HI	NA	F	32 years	No	No	Cholecystectomy	366 / 10.8	NA	NA	NA	NA	[21]
20	AD	c.1739del p.D580Afs*29	Hetero	HI	NA	F	62 years	NA	Recurrent sinusitis, impaired vaccination response to pneumococcus	No	204 / 5.7	Yes	5.60	1.30	0.30	[21]
21	AD	c.1451–18 A>G p.0	Hetero	HI	NA	F	27 years	NA	Pneumonia, bronchitis	Vitiligo	166 / 4.8	Yes	3.90	0.70	0.30	[21]
22	AD	Chr19: 959,646–1,649,320 p.0	Hetero	HI	NA	F	6 years	NA	Recurrent respiratory infections	Crohn’s disease, early onset IBS, Peutz‒Jeghers syndrome, mild motor and intellectual developmental delay	76 / 2.7	Yes	6.46	0.43	0.11	[21]
23^b^	AD	c.503_504insC p.T168Tfs*24	Hetero	HI	NA	F	61 years	Teenage years	Meningitis, chronic diarrhea, recurrent respiratory infections, impaired vaccination response to pneumococcus, diphtheria, tetanus	Hashimoto’s thyroiditis, SLE	NA / NA ↓	Yes	4.50	0.20	0.20	[25]
24	AD	c.503_504insC p.T168Tfs*24	Hetero	HI	NA	M	35 years	NA	Sinusitis, chronic tonsillitis, impaired vaccination response to pneumococcus, diphtheria, tetanus	Type 1 diabetes, seronegative arthritis, synovitis	NA/NA ↓	Yes	5.50	< 0.07	0.40	[25]
25	AD	c.1663 G>A p.E555K	Hetero	DN	North Americans of European	F	34 years	9 months	Pneumococcal meningitis	No	NA / 0.4	Yes	8.59^a^	< 0.07	< 0.07	[18, 19]
26	AD	c.1663 G>A p.E555K	Hetero	DN	North Americans of European	F	8 years	10 months	Recurrent otitis	Eosinophilic dermatitis	NA / 2.5	Yes	< 0.33	0.14	< 0.07	[18, 19]
27	AD	c.1663 G>A p.E555K	Hetero	DN	North Americans of European	M	15 years	2 years	Vaccine-associated polio	Hepatomegaly	NA / 0.2	Yes	8.44^a^	< 0.01	0.10	[18, 19]
28	AD	c.1663 G>A p.E555K	Hetero	DN	Mexican	M	20 years	4 years	Recurrent otitis and arthritis	No	NA / 1.5	Yes	< 1.00	< 0.05	< 0.05	[18, 19]
29	AD	c.1663 G>A p.E555K	Hetero	DN	British	F	35 years	11 months	*Streptococcus pneumoniae* pneumonia and empyema, intermittent sinusitis, *Haemophilus influenzae* conjunctivitis	No	2 / 0.2	Yes	< 0.50	< 0.07	< 0.10	[20]
30	AD	c.1663 G>A p.E555K	Hetero	DN	NA	M	23 years	NA	Gastroenteritis, *Haemophilus influenzae* type B meningitis, otitis, *Rickettsia* infection and pneumonia, bronchiectasis	No	8 / 0.6	Yes	0.33	0.06	0.21	[21]
31	AD	c.1663 G>A p.E555K	Hetero	DN	Japanese	M	25 years	6 months	Recurrent pneumonia, bronchitis, sinusitis, otitis media, mainly due to *Haemophilus influenzae*	Facial dysmorphic features, growth failure with GHD	0 / 0	Yes	0.09	0.01	0.01	This study

^a^ On immunoglobulin therapy, ^b^ The patient also has the heterozygous *TNFRSF13B* variant (c.310 T>C; p.C104R).

*Pt.*, patient; *Ref.*, reference; *AR*, autosomal recessive; *AD*, autosomal dominant; *Homo*, homozygosity; *Hetero*, heterozygosity; *LOF*, loss-of-function; *HI*, haploinsufficiency; *DN*, dominant-negative; *M*, male; *F*, female; *NA*, not available; *B-ALL*, B-cell acute lymphoid leukemia; *EBV*, Epstein‒Barr virus; *IBS*, irritable bowel syndrome; *SLE*, systemic lupus erythematosus; *GHD*, growth hormone deficiency

### Glutamate 555 Is the Key Residue of the E47 bHLH Domain Involved in DNA Binding

**Fig. 2:**
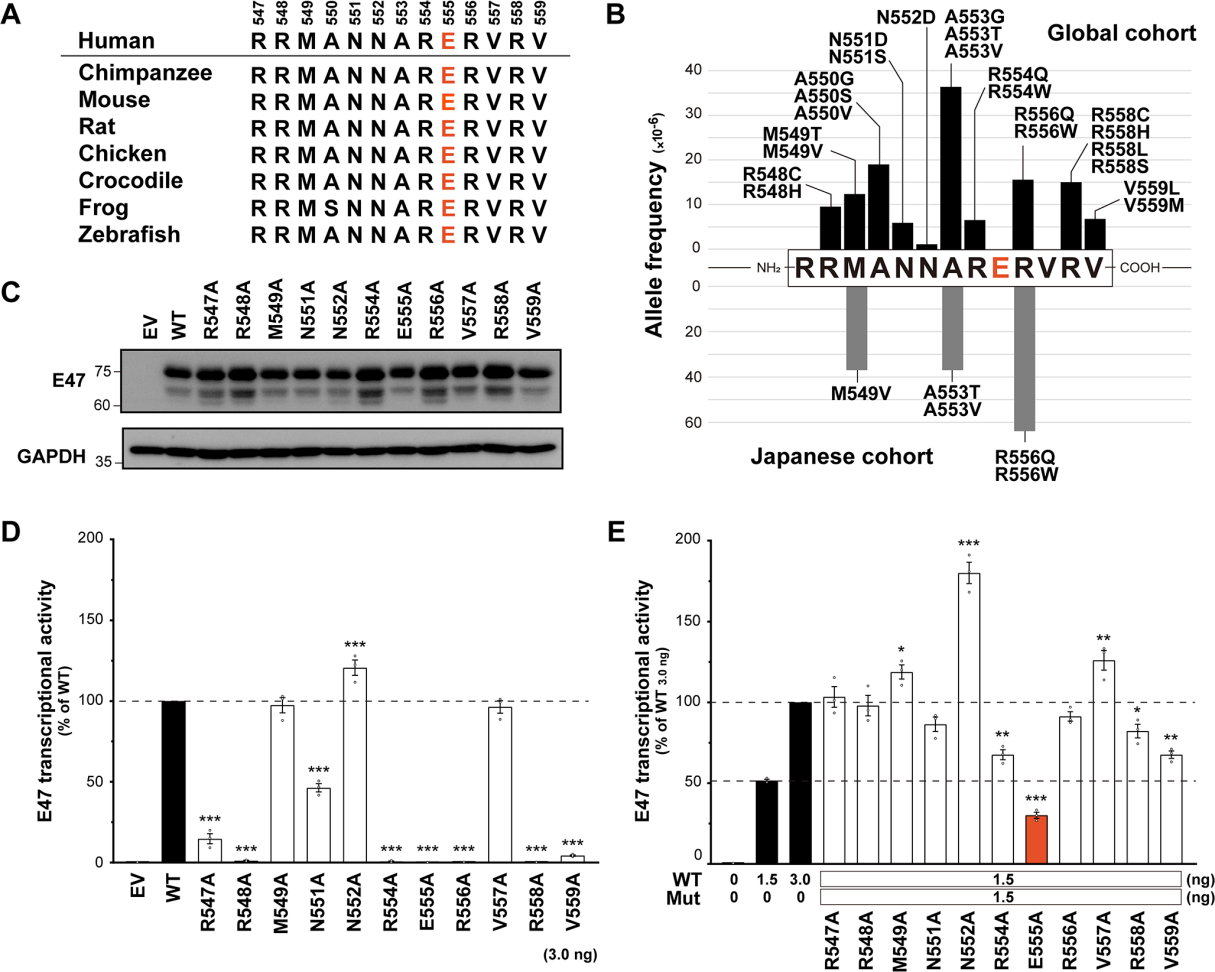
Evaluation of each position in the E47 basic region. (**A**) Conservation of the basic region and the E555 residue among different species. (**B**) Allele frequencies and variant types of the E47 basic region in the general population databases. The global cohort (top) and the Japanese cohort (bottom) are based on the Genome Aggregation Database (gnomAD v4.0.0) and the Tohoku Medical Megabank Organization (ToMMo 54KJPN), respectively. (**C**) Immunoblotting of extracts from human embryonic kidney (HEK) 293T cells transfected with empty vector (EV), E47 wild-type (WT), or alanine variants of the E47 basic region. (**D**) Luciferase reporter assay to mimic the homozygous state in HEK293T cells transfected with EV, WT, or alanine variants. (**E**) Luciferase reporter assay to mimic the heterozygous state in HEK293T cells co-transfected with WT and alanine variants. Statistical analysis was performed using one-way analysis of variance (ANOVA) with Dunnett’s post hoc test compared to WT 3.0 ng (*p < 0.05, **p < 0.01, ***p < 0.0001)

The E47 basic region, including residue E555, is highly conserved among different vertebrate species (Fig. 2A). The allele frequencies of each variant in 13 residues of the E47 basic region were quite rare (the mean allele frequency of the basic region is 10.2 × 10^− 6^) according to global and Japanese population databases, including the Genome Aggregation Database (gnomAD v4.0.0) and the Tohoku Medical Megabank Organization (ToMMo 54KJPN [31]) (Fig. 2B). No variants at residues R547, E555, or V557 were reported in these databases (Fig. 2B). These findings suggest that these positions in the E47 basic region are under selection pressure.

To validate a crucial position in the E47 basic region, we performed alanine scanning of 11 amino acid residues from R547 to V559, excluding A550 and A553. Alanine scanning, which systematically replaces individual amino acid residues in a protein with alanine residues, is a well-known technique for evaluating the role of each residue in protein structure and function [32, 33]. All alanine variants in the basic region were stably expressed in transfected HEK293T cells similar to WT (Fig. 2C). We then evaluated the transcriptional activities of the alanine variants using a luciferase reporter assay. The variants R547A, R548A, N551A, R554A, E555A, R556A, R558A, and V559A showed low or no transcriptional activity, indicating that these variants are LOF (Fig. 2D). In addition, alanine substitutions at M549 and V557, which are not expected to interact with DNA (Fig. S3), did not affect transcriptional activity (Fig. 2D). This finding ensures the reliability of these in vitro experiments. To mimic the heterozygous situation, an equal amount of WT and each alanine variant vector was co-transfected. All alanine variants except E555A showed a recovery of more than the E47 transcriptional activity of WT 1.5 ng (Fig. 2E). These results indicated that the E555A variant is a LOF variant and also has a potential DN effect when co-expressed with the WT. In summary, the E555 residue plays a more important role than other residues in the basic region in binding the DNA motif.

### Impact of the Comprehensive Amino Acid Substitutions Focused on Position 555

**Fig. 3:**
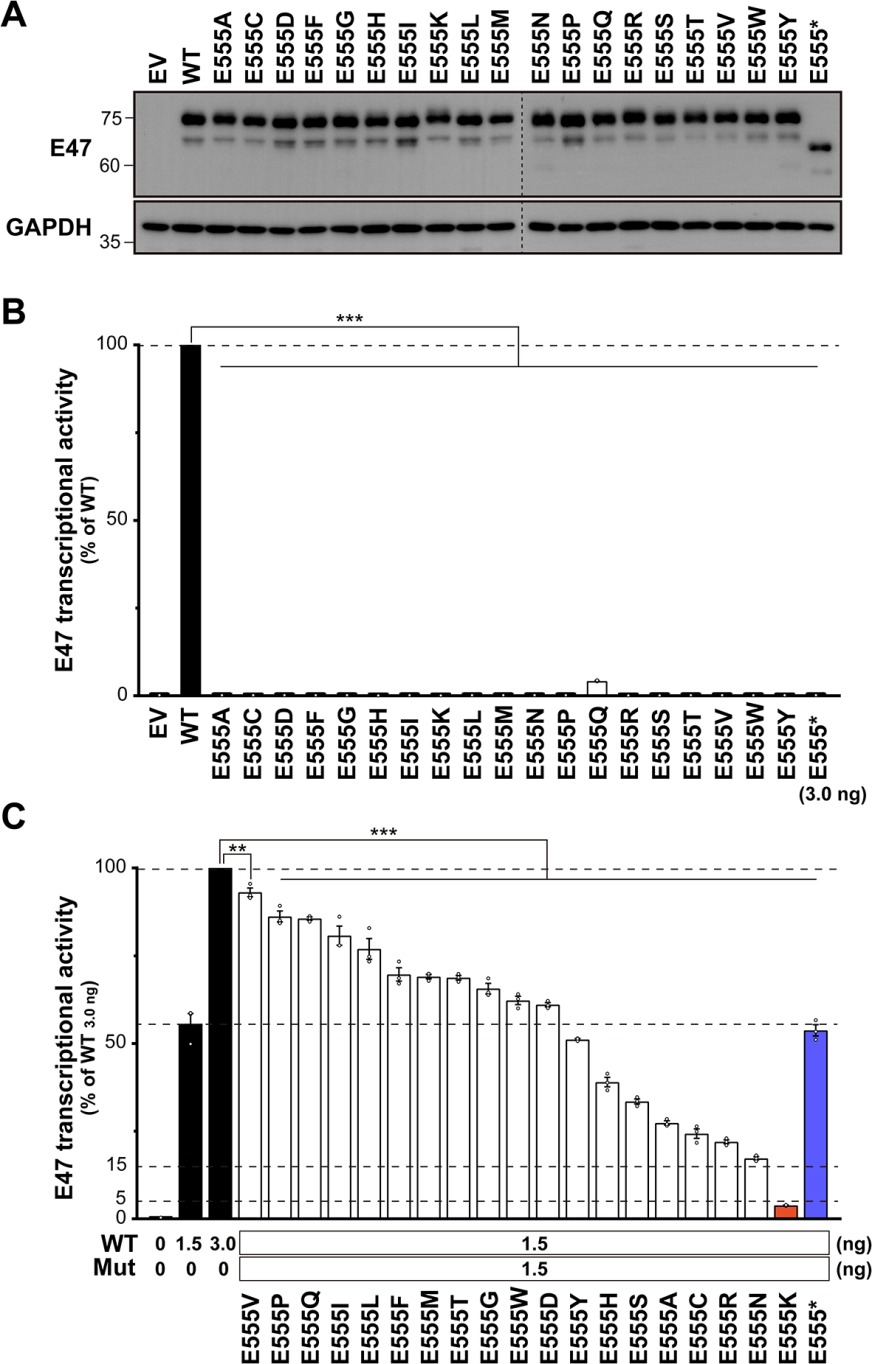
Evaluation of comprehensive amino acid substitutions at position 555. (**A**) Immunoblotting of extracts from human embryonic kidney (HEK) 293T cells transfected with empty vector (EV), E47 wild-type (WT), or E555 variants. (**B**) Luciferase reporter assay to mimic the homozygous state in HEK293T cells transfected with EV, WT, or E555 variants. (**C**) Luciferase reporter assay to mimic the heterozygous state in HEK293T cells co-transfected with WT and E555 variants. Statistical analysis was performed using one-way analysis of variance (ANOVA) with Dunnett’s post hoc test compared to WT 3.0 ng (*p < 0.05, **p < 0.01, ***p < 0.0001)

We then evaluated the effect of single amino acid substitutions at position 555. We generated vectors encoding 20 variants at position 555 that differed from the WT by replacing glutamate with 19 other amino acids or a stop codon. Immunoblot analysis demonstrated the correct expression of both the WT and the designed variants in the transfected HEK293T cells (Fig. 3A). E555* showed a reduced molecular size due to the truncated protein lacking the C-terminal portion, including the bHLH domain (Fig. 3A). Next, we assessed the E47 transcriptional activity of these variants using a luciferase reporter assay, following a procedure similar to the alanine scanning assay. In the case mimicking *TCF3* homozygosity, the transcriptional activity of all E555 variants was significantly reduced, indicating that all single amino acid substitutions at E555 were consistently LOF (Fig. 3B). In contrast, co-transfection of equal amounts of WT vector to mimic *TCF3* heterozygosity restored activity levels to greater than 15% of WT 3.0 ng for almost all variants (Fig. 3C). The only exception was E555K, which remained very low, with activity less than 5% of WT 3.0 ng (Fig. 3C). In addition, the E555Y, E555H, E555S, E555A, E555C, E555R, E555N, and E555K variants had activity less than WT 1.5 ng, suggesting a potential for negative dominance (Fig. 3C).

### Substitution of the E555 Residue with Lysine Significantly Impairs Wild-Type E47 Function

**Fig. 4:**
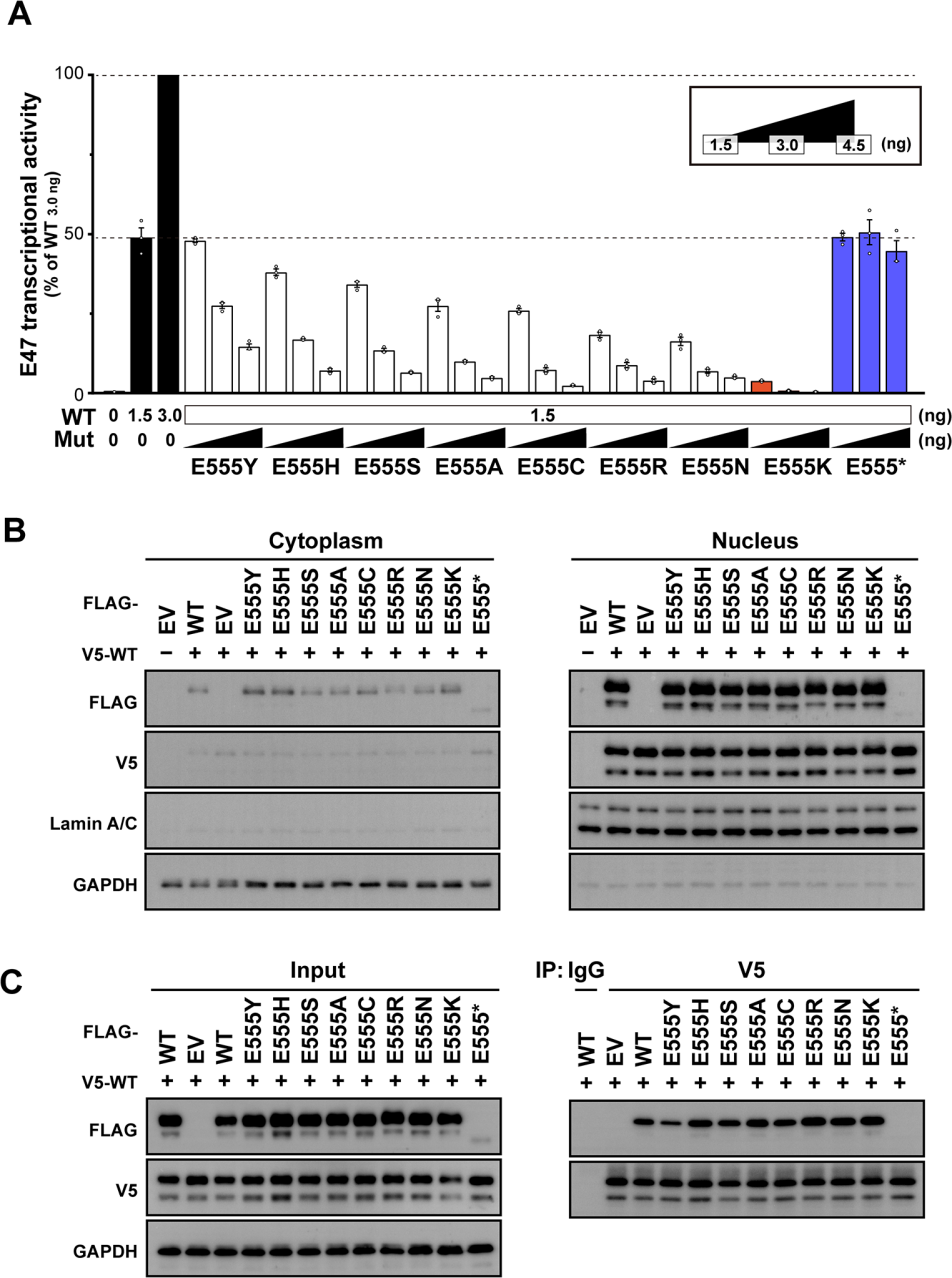
Evaluation of the DN effect of the E555 variants. (**A**) E47 transcriptional activity after co-transfection with the expression vector containing E47 wild-type (WT) (1.5 ng) and each E555 variant (1.5, 3.0, or 4.5 ng). (**B**) Immunoblotting for subcellular E47 protein expression in human embryonic kidney (HEK) 293T cells co-transfected with WT and E555 variants. (**C**) Immunoblot analysis of E47 protein in total protein extracts from HEK293T cells co-transfected with WT and E555 variants (left). Whole-cell lysates were immunoprecipitated with anti-V5 antibody followed by immunoblotting with anti-FLAG or anti-V5 antibody (right). Immunoprecipitation with non-specific IgG was used as an experimental negative control. (**B, C**) Detection of E555* was weaker than in Fig. 3A due to the use of different primary antibodies (anti-human E47 or anti-FLAG antibody) (Fig. S5)

To confirm a DN effect of the E555 variants, we co-transfected a fixed amount (1.5 ng) of the WT vector together with equal (1.5 ng), double (3.0 ng), or triple (4.5 ng) amounts of each variant vector. The E555 variants with negative dominance potential showed relatively strong dose-dependent negative effects, confirming that they are DN (Fig. 4A). Similarly, the other variants without DN potential also showed dose dependence (Fig. S4), which may be due to an increased proportion of non-functional variant dimers compared to partially functional variant‒WT dimers. E555*, which lacks the bHLH domain, had no effect on WT activity, indicating that it is the HI variant. Significantly, E555K exerted an extremely strong negative effect, suggesting that substitution of the E555 residue with lysine has a potent DN effect (Fig. 4A). We then evaluated the subcellular protein fractionation and dimer formation ability to elucidate the driving force behind this potent DN effect. Together with the WT and other DN variants, E555K was predominantly expressed in the nucleus (Fig. 4B) and could form dimers with the WT (Fig. 4C). These results suggest that functional WT is present in the nucleus but is deprived of function by interactions with variants. Despite the drastic DN effect of E555K in the luciferase reporter assay, no clear difference between E555K and the other variants was detected in these qualitative in vitro experiments.

### Structure-Based Variant Analysis

**Fig. 5:**
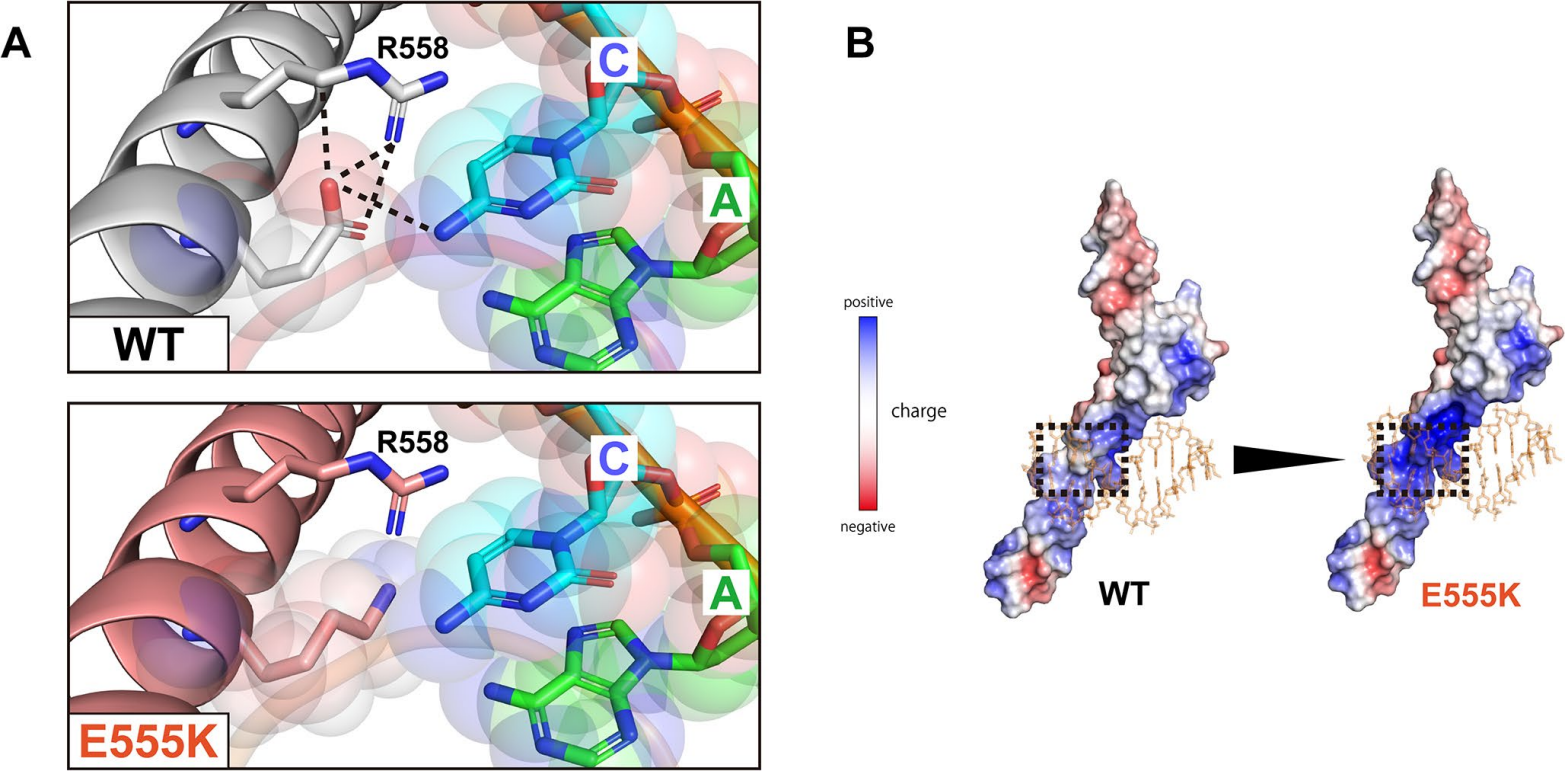
Structural analysis of E47 WT and E555K. (**A**) Structural interaction between E47 proteins and the E-box. The wild-type (WT) E47 protein is shown in gray, and the E555K variant is shown in pale red. Dotted lines represent hydrogen bonds. (**B**) The calculated electrostatic potentials of the WT and E555K variants are superimposed on the surface structures and colored in a gradient from red (negative) to blue (positive)

We performed an in silico analysis to elucidate the molecular pathogenesis of E555K. Using MOE software, we created a structural model of the E555K variant and analyzed its interaction with an E-box. The WT E555 residue was predicted to form hydrogen bonds with the R558 residue and a cytosine base within the E-box (Fig. 5A). In contrast, the variant K555 residue completely disrupted these hydrogen bonds (Fig. 5B). Furthermore, the E555 residue carries a negative charge, whereas the K555 residue carries a positive charge. Electrostatic potential calculations of the protein surface revealed a shift from a neutral to a strongly positive electromagnetic field around position 555 (Fig. 5C). This amino acid substitution was expected to reverse the electrical potential, leading to local repulsion with the specific base and preventing DNA binding. These results strongly suggest that the E555K variant results in LOF. This finding is supported by the previous study that experimentally demonstrated the inability of E555K to bind to the E-box sequence [19].

## Discussion

We report here the first Asian patient with AD E47 transcription factor deficiency caused by the *TCF3* DN variant. The heterozygous *TCF3* DN variant was first shown to be responsible for an AD form of agammaglobulinemia in 2013 [19]. Our patient and 6 previously reported patients consistently have the same variant p.E555K in the E47 basic region [19–21]. In 1990, Voronova and Baltimore [34] experimentally showed that the E47 basic region variants could dimerize with WT E47 but could not bind to the E-box sequence and predicted that these variants were transdominant. However, among the 13 basic region residues, only p.E555K was identified as a pathological variant associated with DN. Therefore, we performed alanine scanning of the E47 basic region and comprehensive mutational analysis focusing on the amino acid residue at position 555 to validate the significance of this position within the basic region and to determine the specificity of the E555K variant. Alanine scanning revealed that 8 out of 11 alanine variants in the basic region were LOF, but only the E555 alanine variant had potential properties for both LOF and DN. This result suggests that the E555 residue is structurally and functionally the most important in the basic region, consistent with previous studies showing that this residue plays a special role in protein‒DNA interactions [35–37]. The alanine variants of the basic region, except E555A, showed higher transcriptional activity than WT 1.5 ng when co-expressed with WT. These variants may not be pathogenic, at least in the heterozygous state, supporting multiple reports of variants in the E47 basic region in public databases. In addition, comprehensive mutational analysis focused on position 555 revealed that all E555 variants were LOF, but the negative dominance effect over WT E47 varied depending on the type of amino acid substitution. Intriguingly, the E555K variant had the most potent DN effect among all E555 variants. This result partially explains why only p.E555K has been identified as the *TCF3* DN variant to date.

From the protein structure prediction, it was shown that the substitution of glutamate with lysine at position 555 disrupts all hydrogen bonds with the E-box base and with residue R558. We noted the conflicting electrical properties of glutamate and lysine, and calculated the electrostatic potential of the E47 protein surface. As expected, this substitution led to a drastic electromagnetic change of the protein surface from neutral to strongly positive. Protein surface charges generate electrostatic interactions and play an essential role not only in protein‒DNA but also in protein‒protein interactions [38]. These molecular properties of protein side chains have been used to study artificial protein engineering to obtain more stable dimers [39, 40]. Similarly, the E555K‒WT interaction may be more stabilized than WT‒WT or E555K‒E555K under the influence of this electromagnetic field. This hypothesis is very compelling to explain the pronounced DN effect of E555K. However, our in vitro experiments, including the co-immunoprecipitation assay, showed no discernible difference between E555K and the other DN variants. Further precise and quantitative evaluation is needed to elucidate the mechanism underlying the pronounced DN effect of E555K.

The clinical features of AD E47 transcription factor deficiency are a severe reduction of B cells and agammaglobulinemia. Our patient presented with mild facial dysmorphism and growth failure in addition to typical B-cell deficiency and agammaglobulinemia. These additional features have not been reported in previously identified patients with AD E47 transcription factor deficiency, but similar phenotypes have been reported in several patients with AR TCF3 deficiency (Table 1) [21, 22]. The *TCF3* DN variant may also be associated with these non-immunological symptoms. However, it should be noted that they may be caused by independent genetic variants not identified in our IEI-related gene panel or by synergistic interactions between loci known as epistasis. Furthermore, the complete loss of TCF3 proteins, including E12 and E47, is a potential risk for the development of B-ALL [21, 22]. B-ALL is a life-threatening disease, and 1 patient with AR TCF3 deficiency has died as a result of this disease. Fortunately, B-ALL has not been reported in patients with AD E47 deficiency (Table 1) [18–21]. A recent systematic study suggested that *TCF3* E12 germline variants alter B-cell maturation, which may increase the risk of preleukemic clone emergence [41]. The susceptibility to develop B-ALL may depend on the retention of functional E12 transcription factors. Although some clinical manifestations of TCF3 deficiency may be common to both inheritance traits, additional cases are needed to determine whether these symptoms are characteristic of the *TCF3* DN variant.

Inborn errors of immunity-related genes with AD inheritance cause severe disease and are generally expected to be the primary targets of strong negative selection. However, the *TCF3* locus has a weak negative selection score (CoNeS of 0.420), which is unusual for an IEI-related gene with dominant traits of HI or DN [42]. The heterogeneity in the selective constraints on *TCF3* causes this weak selective constraint; almost all domains of this gene are not particularly constrained (subRVIS score of 83.8%), whereas the bHLH domain is under relatively strong negative selection (subRVIS score of 18.4%) [42, 43]. Furthermore, variant effect prediction using protein language models highlights that the bHLH domain contains many vulnerable positions, which may be a driving force behind the negative selection for this domain (Fig. S6) [44]. Our comprehensive study experimentally demonstrated that in the homozygous state, there are multiple mutation-sensitive sites in the E47 basic region. Conversely, in the heterozygous state, the mutation-sensitive site was restricted to position 555 alone, and more interestingly, the transcriptional activity varied widely depending on the type of amino acid substitution. The heterogeneity in the selective constraint on *TCF3*, at least in an AD trait, may be determined at the residue rather than the domain level.

In conclusion, we report the first Asian patient with AD E47 transcription factor deficiency caused by the heterozygous *TCF3* variant. Our comprehensive functional analysis partially elucidates the exclusivity of the p.E555K variant and the weak negative selection of *TCF3*. However, further studies are necessary to understand the full picture of this unique gene.

### Electronic Supplementary Material

Below is the link to the electronic supplementary material.

Supplementary Material 1

## Data Availability

No datasets were generated or analysed during the current study.

## Abbreviations

AD: Autosomal dominant
AR: Autosomal recessive
B-ALL: B-cell acute lymphoblastic leukemia
bHLH: Basic helix-loop-helix
CVID: Common variable immunodeficiency
DN: Dominant-negative
HI: Haploinsufficiency
IEI: Inborn errors of immunity
IVIG: Intravenous immunoglobulin
LOF: Loss-of-function
NK: Natural killer
PBMCs: Peripheral blood mononuclear cells
TCF3: Transcription factor 3
WT: Wild-type
