# Correction to: Exclusive Characteristics of the p.E555K Dominant-Negative Variant in Autosomal Dominant E47 Deficiency

**DOI:** 10.1007/s10875-024-01785-8

**Published:** 2024-08-15

**Authors:** Takanori Utsumi, Miyuki Tsumura, Masato Yashiro, Zenichiro Kato, Kosuke Noma, Fumiaki Sakura, Reiko Kagawa, Yoko Mizoguchi, Shuhei Karakawa, Hidenori Ohnishi, Charlotte Cunningham-Rundles, Peter D. Arkwright, Masao Kobayashi, Hirokazu Kanegane, Dusan Bogunovic, Bertrand Boisson, Jean-Laurent Casanova, Takaki Asano, Satoshi Okada

**Affiliations:** 1 Department of Pediatrics, Graduate School of Biomedical and Health Sciences, Hiroshima University, Hiroshima, Japan; 2 Department of Pediatrics, Okayama University Hospital, Okayama, Japan; 3 Department of Pediatrics, Graduate School of Medicine, Gifu University, Gifu, Japan; 4 Structural Medicine, United Graduate School of Drug Discovery and Medical Information Science, Gifu University, Gifu, Japan; 5 Department of Applied Genomics, Kazusa DNA Research Institute, Chiba, Japan; 6 Division of Allergy and Clinical Immunology, Departments of Medicine and Pediatrics, Icahn School of Medicine at Mount Sinai, New York, NY USA; 7 Lydia Becker Institute of Immunology and Inflammation, University of Manchester, Manchester, UK; 8 Japanese Red Cross Chugoku-Shikoku Block Blood Center, Hiroshima, Japan; 9 Department of Child Health and Development, Graduate School of Medical and Dental Sciences, Tokyo Medical and Dental University (TMDU), Tokyo, Japan; 10 Center for Inborn Errors of Immunity, Icahn School of Medicine at Mount Sinai, New York, NY USA; 11 Precision Immunology Institute, Icahn School of Medicine at Mount Sinai, New York, NY USA; 12 Icahn School of Medicine at Mount Sinai, Mindich Child Health and Development Institute, New York, NY USA; 13 Department of Pediatrics, Icahn School of Medicine at Mount Sinai, New York, NY USA; 14 Department of Microbiology, Icahn School of Medicine at Mount Sinai, New York, NY USA; 15 St. Giles Laboratory of Human Genetics of Infectious Diseases, Rockefeller Branch, The Rockefeller University, New York, NY USA; 16 Laboratory of Human Genetics of Infectious Diseases, Necker Branch, INSERM U1163, Necker Hospital for Sick Children, Paris, France; 17 Imagine Institute, Paris Descartes University, Paris, France; 18 Pediatric Hematology-Immunology Unit, Necker Hospital for Sick Children, AP-HP, Paris, France; 19 Howard Hughes Medical Institute (HHMI), New York, NY USA; 20 Department of Genetics and Cell Biology, Research Institute for Radiation Biology and Medicine, Hiroshima University, Hiroshima, Japan

**Correction to: Journal of Clinical Immunology (2024) 44:167**

10.1007/s10875-024-01758-x

The wrong Supplementary file was originally published with this article; it has now been replaced with the correct file.

The original version has been corrected.

