# Supplementary material for: Exclusive Characteristics of the p.E555K Dominant-Negative Variant in Autosomal Dominant E47 Deficiency
Source: J Clin Immunol. 2024 Jul 29;44(7):167. doi: 10.1007/s10875-024-01758-x (PMC11286708; doi:10.1007/s10875-024-01758-x)
Supplement: Supplementary file 1 — Supplementary Material 1 [file 10875_2024_1758_MOESM1_ESM.docx]

**Supplementary Material**

**Exclusive Characteristics of the p.E555K Dominant-Negative Variant in Autosomal Dominant E47 Deficiency**

**Authors**

Takanori Utsumi^1^, Miyuki Tsumura^1^, Masato Yashiro^2^, Zenichiro Kato^3,4^, Kosuke Noma^1^, Fumiaki Sakura^1,5^, Reiko Kagawa^1^, Yoko Mizoguchi^1^, Shuhei Karakawa^1^, Hidenori Ohnishi^3^, Charlotte Cunningham-Rundles^6^, Peter D. Arkwright^7^, Masao Kobayashi^8^, Hirokazu Kanegane^9^, Dusan Bogunovic^10, 11, 12, 13, 14^, Bertrand Boisson^15, 16, 17^, Jean-Laurent Casanova^15, 16, 17, 18, 19^, Takaki Asano^1, 20^*, Satoshi Okada^1^*

**Affiliations**

^1^ Department of Pediatrics, Graduate School of Biomedical and Health Sciences, Hiroshima University, Hiroshima, Japan

^2^ Department of Pediatrics, Okayama University Hospital, Okayama, Japan

^3^ Department of Pediatrics, Graduate School of Medicine, Gifu University, Gifu, Japan

^4^ Structural Medicine, United Graduate School of Drug Discovery and Medical Information Science, Gifu University, Gifu, Japan

^5^ Department of Applied Genomics, Kazusa DNA Research Institute, Chiba, Japan

^6^ Division of Allergy and Clinical Immunology, Departments of Medicine and Pediatrics, Icahn School of Medicine at Mount Sinai, New York, NY, USA

^7^ Lydia Becker Institute of Immunology and Inflammation, University of Manchester, Manchester, UK

^8^ Japanese Red Cross Chugoku-Shikoku Block Blood Center, Hiroshima, Japan

^9^ Department of Child Health and Development, Graduate School of Medical and Dental Sciences, Tokyo Medical and Dental University (TMDU), Tokyo, Japan

^10^ Center for Inborn Errors of Immunity, Icahn School of Medicine at Mount Sinai, New York, NY, USA

^11^ Precision Immunology Institute, Icahn School of Medicine at Mount Sinai, New York, NY, USA

^12^ Mindich Child Health and Development Institute, Icahn School of Medicine at Mount Sinai, New York, NY, USA

^13^ Department of Pediatrics, Icahn School of Medicine at Mount Sinai, New York, NY, USA

^14^ Department of Microbiology, Icahn School of Medicine at Mount Sinai, New York, NY, USA

^15^ St. Giles Laboratory of Human Genetics of Infectious Diseases, Rockefeller Branch, The Rockefeller University, New York, NY, USA

^16^ Laboratory of Human Genetics of Infectious Diseases, Necker Branch, INSERM U1163, Necker Hospital for Sick Children, Paris, France

^17^ Paris Descartes University, Imagine Institute, Paris, France

^18^ Pediatric Hematology-Immunology Unit, Necker Hospital for Sick Children, AP-HP, Paris, France

^19^ Howard Hughes Medical Institute (HHMI), New York, NY, USA

^20^ Department of Genetics and Cell Biology, Research Institute for Radiation Biology and Medicine, Hiroshima University, Hiroshima, Japan

***Corresponding authors**

Takaki Asano

Department of Pediatrics, Graduate School of Biomedical and Health Sciences, Hiroshima University, Hiroshima, Japan, 734-8551, Tel: +81-82-257-5212 Fax: +81-82-257-5214

Department of Genetics and Cell Biology, Research Institute for Radiation Biology and Medicine, Hiroshima University, Hiroshima, Japan, 734-8551, Tel: +81-82-257-5811

E-mail: [tasano02@hiroshima-u.ac.jp](mailto:tasano02@hiroshima-u.ac.jp)

Satoshi Okada

Department of Pediatrics, Graduate School of Biomedical and Health Sciences, Hiroshima University, Hiroshima, Japan, 734-8551, Tel: +81-82-257-5212 Fax: +81-82-257-5214

E-mail: [sokada@hiroshima-u.ac.jp](mailto:sokada@hiroshima-u.ac.jp)

**This file includes:**

**Supplementary Methods**

**Supplementary Table 1**

**Supplementary Figures S1, S2, S3, S4, S5, S6**

**Supplementary Methods**

**Flow cytometry analysis**

Multicolor flow cytometric analysis was performed as previously described [30]. Peripheral blood mononuclear cells (PBMCs) were isolated from whole blood by density gradient centrifugation using Lymphoprep (Axis-Shield Diagnostics Limited, Dundee, Scotland, UK). The lymphocyte subset was stained with each monoclonal antibody corresponding to the subset marker. We performed multicolor flow cytometry using a BD LSRFortessa (BD Biosciences, Franklin Lakes, NJ, USA) and analyzed the results using FlowJo software (FlowJo LLC, Ashland, OR, USA).

**Immunoblot analysis of peripheral blood mononuclear cells**

Frozen PBMCs were thawed, and then approximately 1.0 × 10^6^ cells/well in a 24-well plate were cultured in RPMI1640 (Thermo Fisher Scientific, Waltham, MA, USA) containing 10% heat-inactivated fetal bovine serum supplemented with 100 μg/mL penicillin/streptomycin overnight at 37°C in the presence of 5% CO_2_. Cells were lysed in RIPA lysis buffer (Sigma‒Aldrich, St. Louis, MO, USA) plus protease and phosphatase inhibitor cocktails (Thermo Fisher Scientific). Whole-cell protein extracts were separated by SDS‒PAGE and transferred to polyvinylidene fluoride membranes (Merck KGaA, Darmstadt, Germany). The membrane was blocked with 10% skim milk (Becton Dickinson, Franklin Lakes, NJ, USA) for 60 minutes at room temperature and then incubated overnight at 4°C with mouse anti-human E47 antibody (1:500 dilution; RRID: AB_395228, BD Biosciences, San Jose, CA, USA) and mouse anti-GAPDH antibody (1:1,000 dilution; RRID: AB_1078991, Sigma‒Aldrich) as primary antibodies. Horseradish peroxidase-conjugated anti-mouse antibody (1:2,000 dilution; RRID: AB_772210, Cytiva, Malborough, MA, USA) was used as a secondary antibody. Antibody binding was detected by chemiluminescence using an ImmunoStar Zeta or LD (Fujifilm Wako Pure Chemical Corporation, Osaka, Japan).

**Table S1: Deep immunophenotyping of the patient’s peripheral blood mononuclear cells**

|  | Patient | Reference [30]  Mean ± SD |
| --- | --- | --- |
| **T cells** (% of lymphocytes) | 79.7 | 67.8 ± 5.4 |
| CD4^+^ T cells (% of CD3^+^) | 49.2 | 59.9 ± 9.9 |
| Naïve CD4^+^ cells (% of CD3^+^CD4^+^) | 72.9 | 47.2 ± 9.3 |
| TCM CD4^+^ cells (% of CD3^+^CD4^+^CD45RO^+^) | 39.2 | 30.9 ± 7.9 |
| TEM CD4^+^ cells (% of CD3^+^CD4^+^CD45RO^+^) | 25.2 | 30.9 ± 7.9 |
| CD8^+^ T cells (% of CD3^+^) | 33.1 | 34.1 ± 8.7 |
| Naïve CD8^+^ cells (% of CD3^+^CD8^+^) | 54.5 | 64.2 ± 7.1 |
| TCM CD8^+^ cells (% of CD3^+^CD8^+^CD45RO^+^) | 2.64 | 11.3 ± 5.2 |
| TEM CD8^+^ cells (% of CD3^+^CD8^+^CD45RO^+^) | 78.7 | 62.0 ± 10.8 |
| iNKT cells (% of CD3^+^) | 0.002 | 0.018 ± 0.012 |
| RTEs (% of CD3^+^CD4^+^CD45RO^+^) | 43.4 | 73.7 ± 11.2 |
| αβT cells (% of CD3^+^) | 81.0 | 89.6 ± 4.8 |
| γδT cells (% of CD3^+^) | 18.6 | 5.2 ± 4.2 |
| DNT cells (% of CD3^+^TCRαβ^+^) | 2.15 | 0.77 ± 0.35 |
| Treg cells (% of CD3^+^CD4^+^) | 4.51 | 3.11 ± 1.02 |
| Tfh cells (% of CD3^+^CD4^+^) | 1.80 | 7.02 ± 3.43 |
| Th1 cells (% of CD3^+^CD4^+^CD45RO^+^) | 19.0 | 22.6 ± 8.7 |
| Th2 cells (% of CD3^+^CD4^+^CD45RO^+^) | 32.5 | 35.3 ± 13.8 |
| Th17 cells (% of CD3^+^CD4^+^CD45RO^+^) | 25.5 | 23.7 ± 4.3 |
| Activated T cells (% of CD3^+^CD4^+^) | 1.39 | 5.18 ± 3.14 |
|  |  |  |
| **B cells** (% of lymphocytes) | 0.0 | 12.2 ± 4.4 |
| CD21^−^ B cells (% of CD20^+^) | NA^*^ | 14.3 ± 5.6 |
| Memory B cells (% of CD19^+^) | NA^*^ | 18.5 ± 8.2 |
| Transitional B cells (% of CD19^+^) | NA^*^ | 8.1 ± 6.5 |
| Plasmablasts (% of CD19^+^) | NA^*^ | 3.22 ± 2.33 |
| IgM memory B cells (% of CD19^+^) | NA^*^ | 11.16 ± 4.01 |
| Switched memory B cells (% of CD19^+^) | NA^*^ | 13.22 ± 7.23 |
| IgG^+^ memory B cells (% of CD19^+^) | NA^*^ | 2.40 ± 1.36 |
| IgA^+^ memory B cells (% of CD19^+^) | NA^*^ | 3.33 ± 2.78 |
|  |  |  |
| **NK cells** (% of lymphocytes) | 7.8 | 13.4 ± 4.1 |

^*^ Could not be evaluated due to the lack of B cells.

The red characters represent values above the reference mean +2 SD. The blue characters represent values below the reference mean −2 SD. *SD,* standard deviation; *TCM*, central memory; *TEM*, effector memory; *iNKT*, invariant natural killer T; *RTEs*, recent thymic emigrants; *Treg*, regulatory T; *Tfh*, follicular helper T; *NK*, natural killer; *NA*, not available.

**Fig. S1: Cytograms of deep immunophenotyping with multicolor flow cytometry**


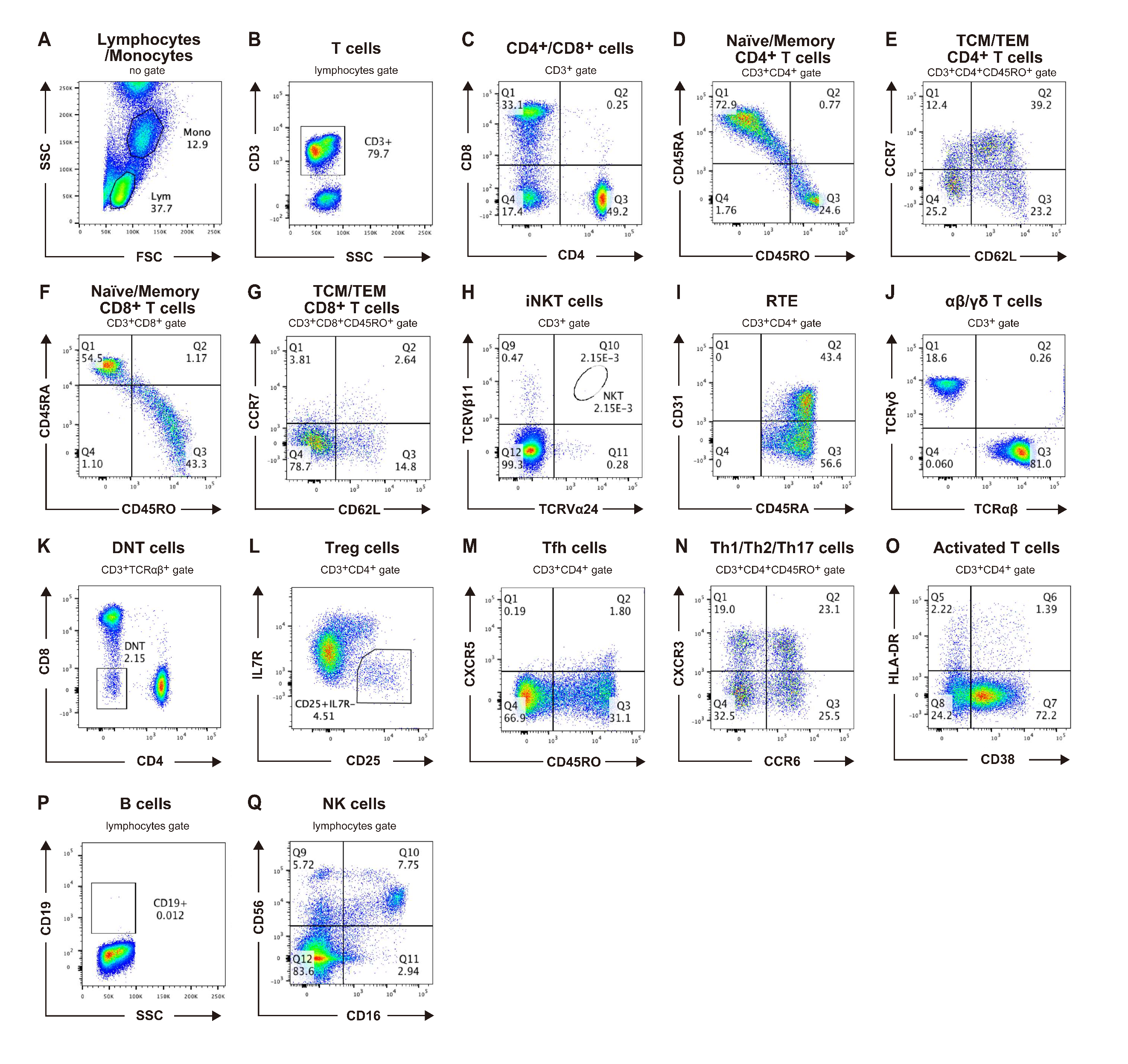


Identification of lymphocyte and monocyte population (A). The proportion of T (CD3^+^) cells in the lymphocyte population (B). The proportions of CD4^+^ or CD8^+^ T cells among the total CD3^+^ cells (C). The proportions of naïve (CD45RO^−^CD45RA^+^) and memory (CD45RO^+^CD45RA^−^) cells among the CD4^+^ T cells (D). The proportions of TCM (CD62L^+^CCR7^+^) and TEM (CD62L^−^CCR7^−^) cells among the CD4^+^ memory T cells (E). The proportion of naïve (CD45RO^−^CD45RA^+^) or memory (CD45RO^+^CD45RA^−^) cells among the CD8^+^ T cells (F). The proportion of TCM (CD62L^+^CCR7^+^) or TEM (CD62L^−^CCR7^−^) cells among the CD8^+^ memory T cells (G). The proportion of iNKT cells (TCRVα24^+^TCRVβ11^+^) among the total CD3^+^ cells (H). The proportion of RTEs (CD31^+^) among the CD4^+^ naïve T cells (I). The proportions of αβT cells (TCRαβ^+^TCRγδ^−^) and γδT cells (TCRαβ^−^TCRγδ^+^) among the total CD3^+^ cells (J). The proportion of DNT cells (CD4^−^CD8^−^) among the αβT cells (K). The proportion of Treg cells (CD25^+^IL7R^−^) among the CD4^+^ T cells (L). The proportion of Tfh cells (CD45RO^+^CXCR5^−^) among the CD4^+^ T cells (M). The proportions of Th1 (CCR6^−^CXCR3^+^), Th2 (CCR6^−^CXCR3^−^), and Th17 (CCR6^+^CXCR3^−^) cells among the CD4^+^ memory T cells (N). The proportion of activated T (CD38^+^HLA-DR^+^) cells among the CD4^+^ T cells (O). The proportion of B (CD19^+^) cells in the lymphocyte population (P). The proportion of NK cells in the lymphocyte population (Q). *TCM*, central memory; *TEM*, effector memory; *iNKT*, invariant natural killer T; *RTEs*, recent thymic emigrants; *Treg*, regulatory T; *Tfh*, follicular helper T; *NK*, natural killer.


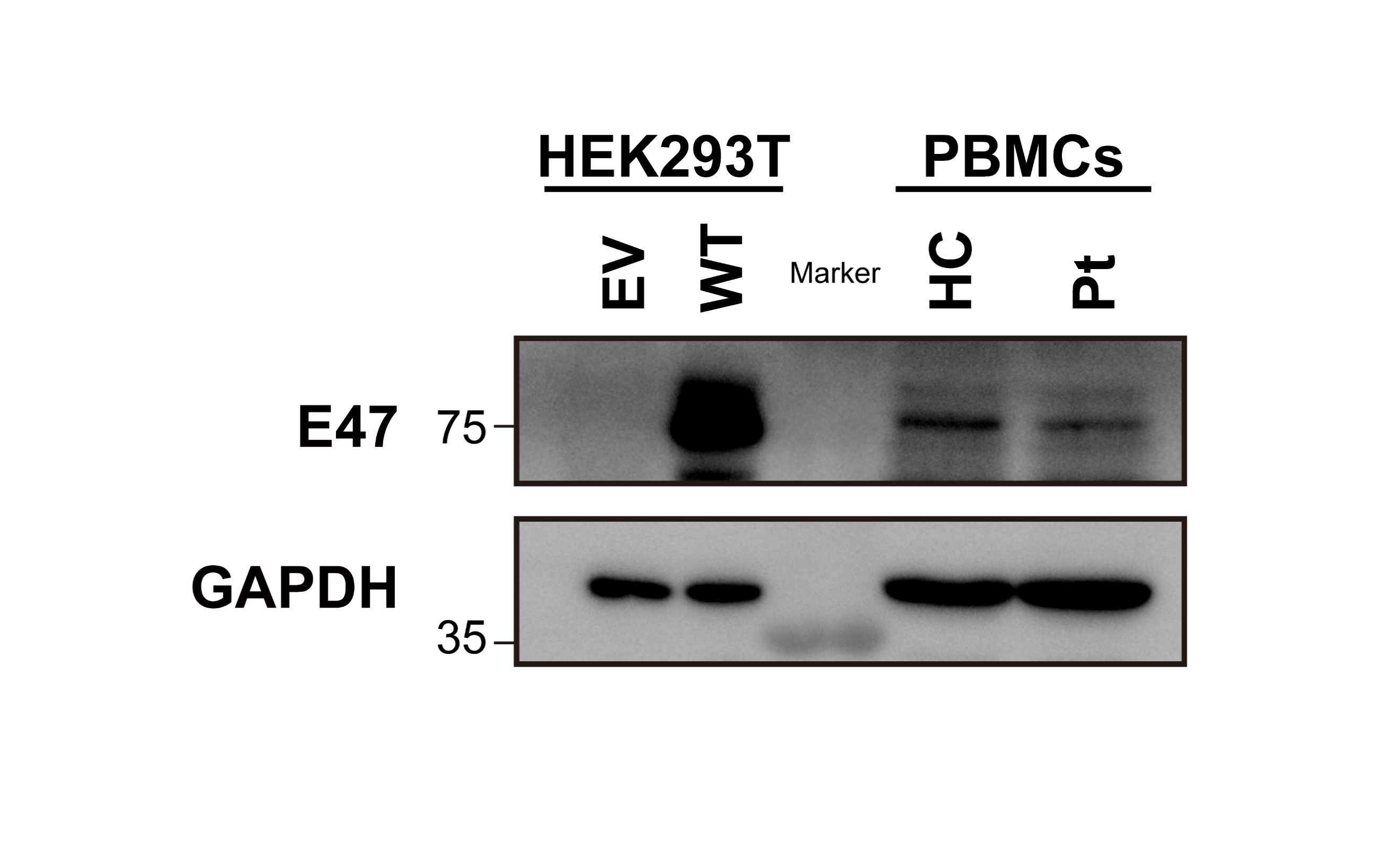
**Fig. S2: E47 protein expression** **in the patient’s peripheral blood mononuclear cells**

Immunoblotting of whole-cell extracts from the human embryonic kidney (HEK) 293T cells transfected with empty vector (EV) or vector encoding with E47 wild-type (WT) or peripheral blood mononuclear cells (PBMCs) of the healthy control (HC) and the patient (Pt).

**Fig. S3: Evaluation of the proximity of residues in the basic region to the E-box**


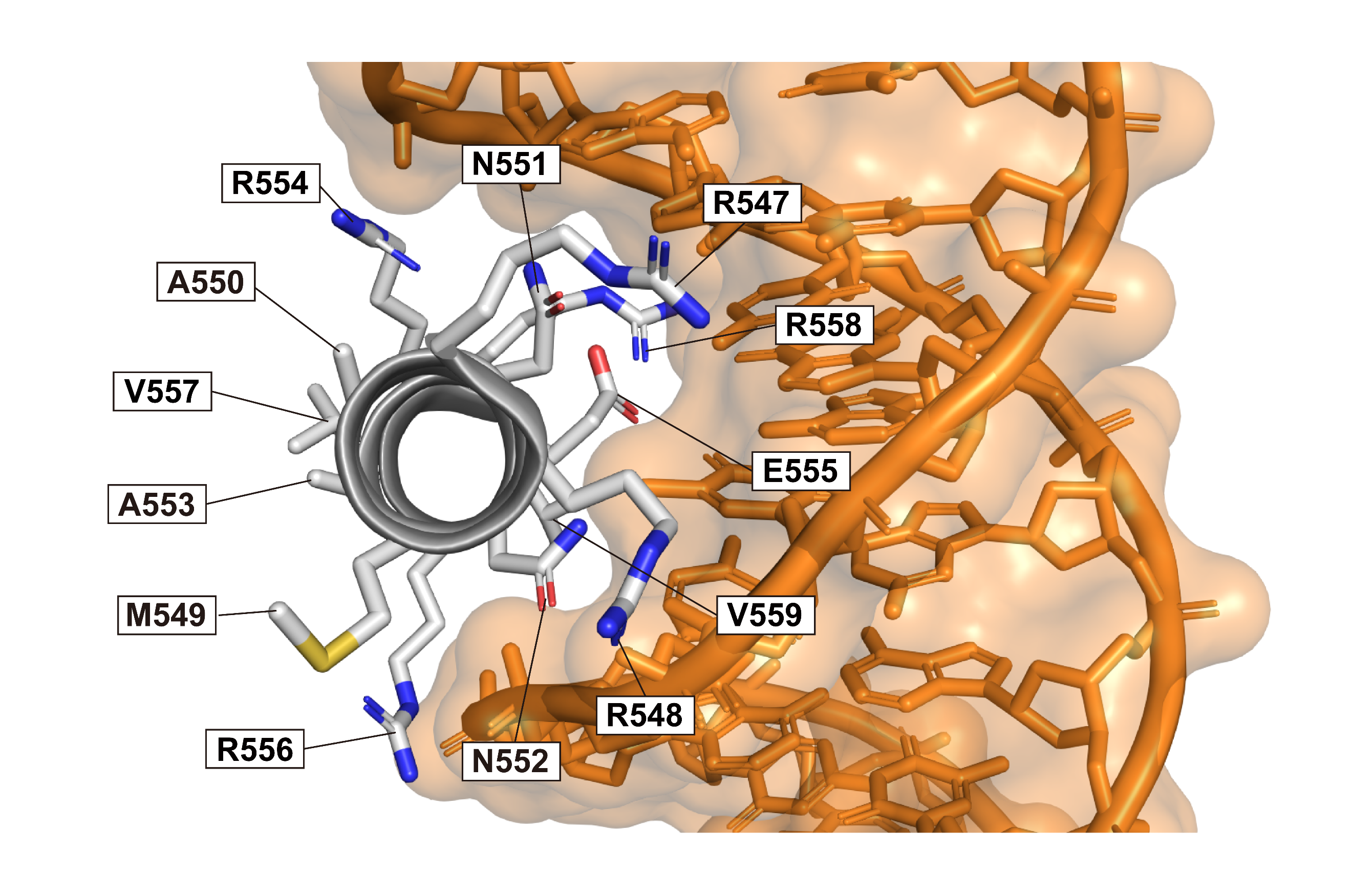


The basic region and E-box structure model were visualized using PyMOL from the human SCL:E47 heterodimer data (PDB ID: 2YPB). Residues M549, A550, A553, and V557 are opposite the E-box.

**Fig. S4: Validation of the DN effect of all E555 variants**


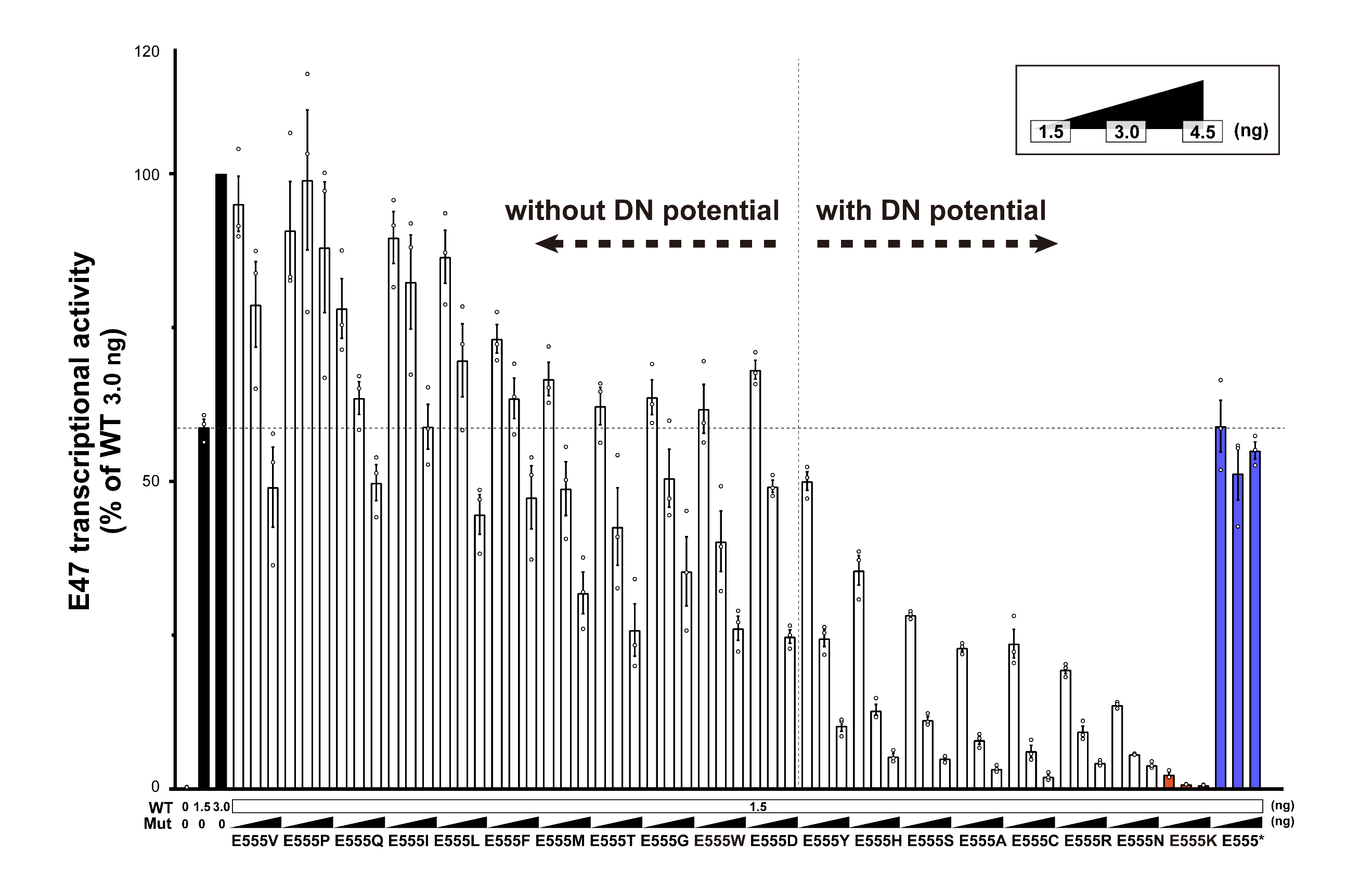
E47 transcriptional activity after co-transfection with the expression vector containing E47 wild-type (WT) (1.5 ng) and each E555 variant (1.5, 3.0, or 4.5 ng). All E555 variants showed a dose-dependent negative effect. However, the variants of E555V, E555P, E555Q, E555I, E555L, E555F, E555M, E555T, E555G, E555W, and E555D are considered to have no dominant-negative (DN) potential because these variants cannot suppress the WT activity when co-transfected with an equal amount of WT.


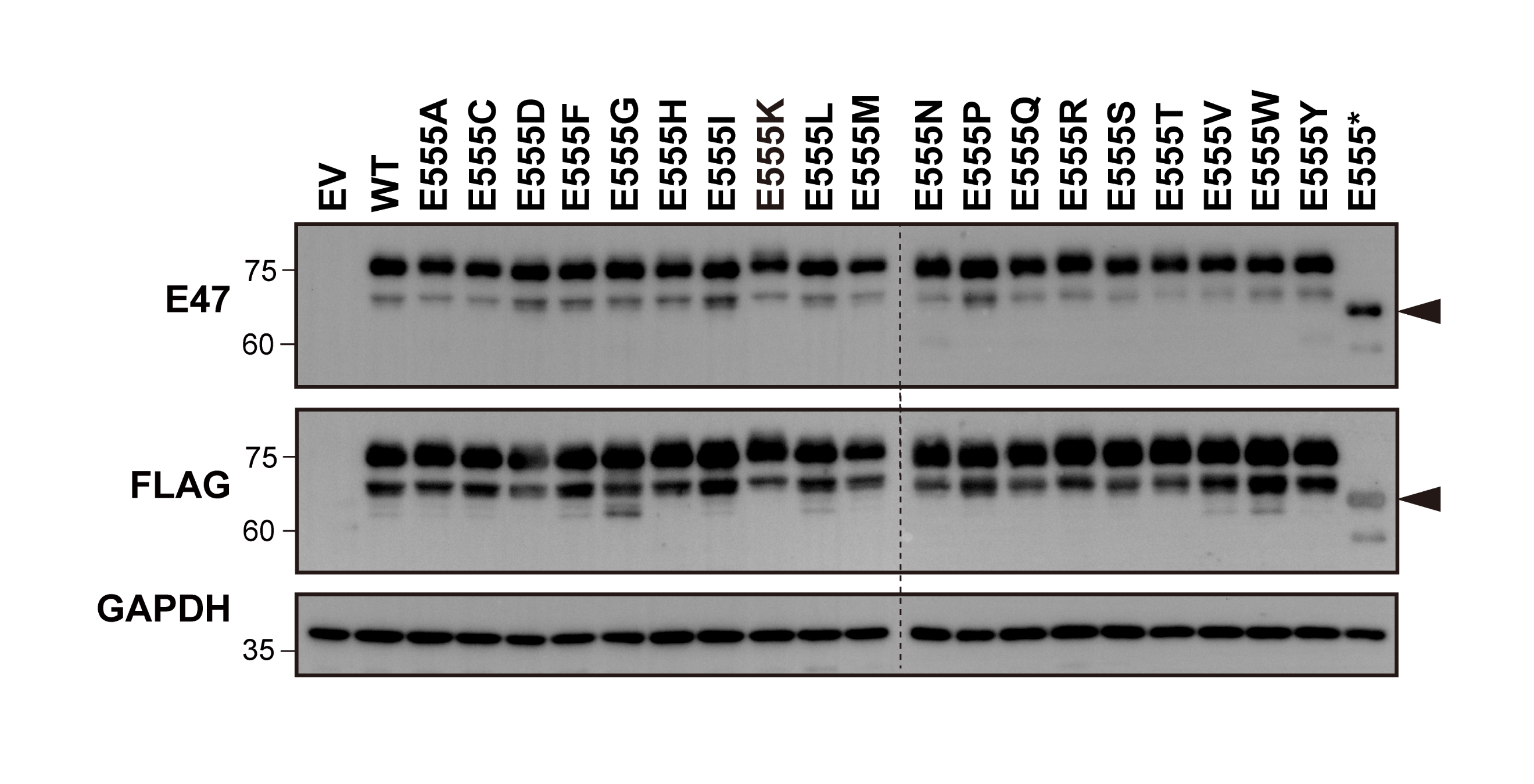
**Fig. S5: The difference in detection of E555* by primary antibodies**

Immunoblotting of whole-cell extracts from human embryonic kidney (HEK) 293T cells transfected with empty vector (EV), E47 wild-type (WT), or E555 variants. E47 was detected with primary antibodies against anti-human E47 antibody (top) and anti-FLAG antibody (middle). Black triangles represent E555* bands. The sensitivity of the FLAG antibody to detect the E555* variant was lower than that of the E47 antibody.


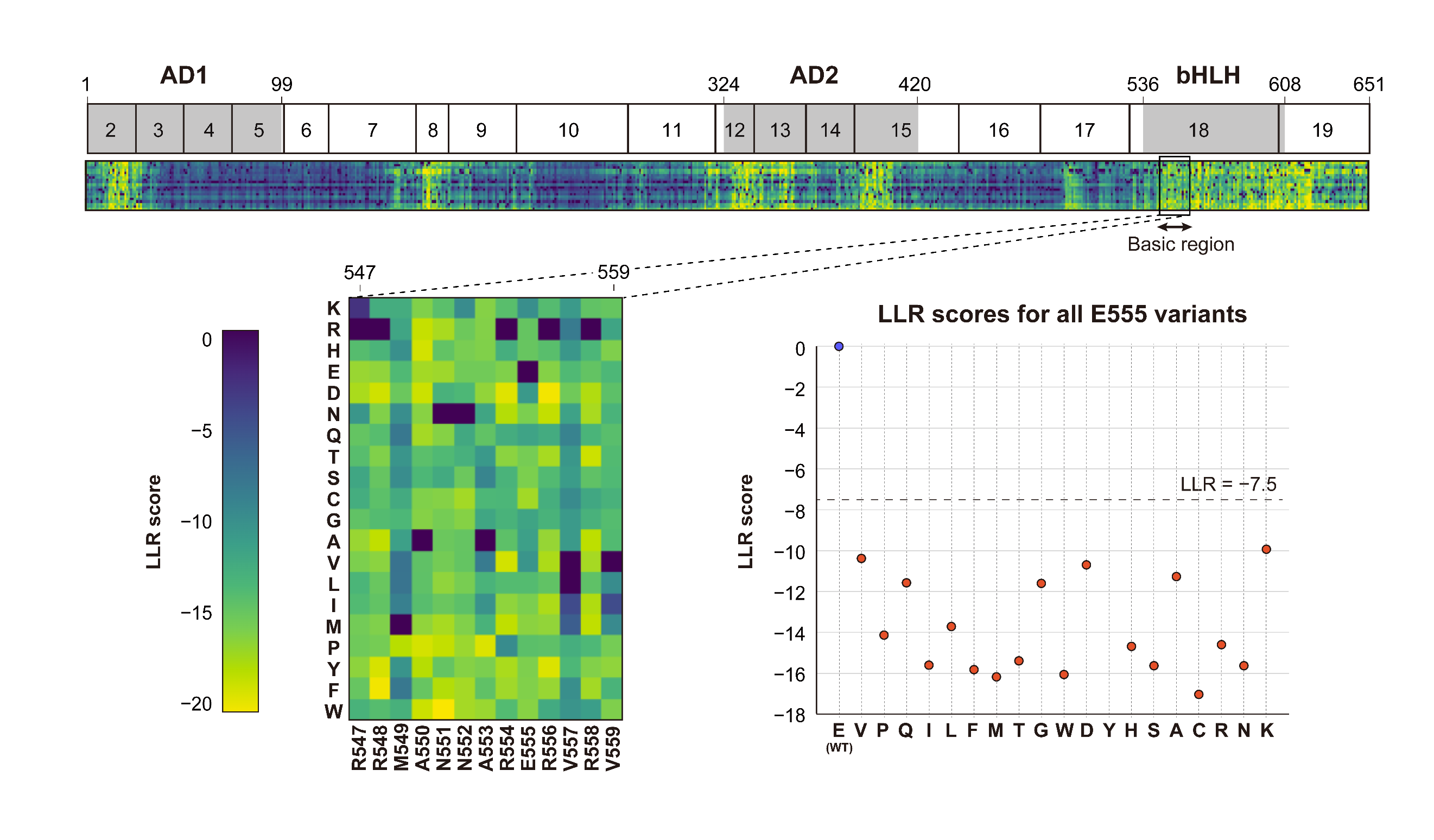
**Fig. S6: Variant effect prediction of all possible missense variants for the E47 protein**

Missense variant effect prediction for the E47 protein using a protein language model, EMS1b (<https://huggingface.co/spaces/ntranoslab/esm_variants>) [44]. Schematic diagram of the E47 protein domain and heat map visualizing the variant effect scores for all possible missense variants as log-likelihood ratios (LLR) between the variant and wild-type (WT) (top). The LLR scores close to 0 indicate benign (blue), while more negative effect scores indicate deleterious (yellow). Protein regions with many possible variants predicted to be damaging matched well with the E47 domains: activation domains 1 and 2 (AD1 and AD2) and basic helix-loop-helix domain (bHLH). The LLR heat map of the E47 basic region (lower left). The plot of LLR scores for all E555 variants (lower right). The black dashed line represents -7.5 of the LLR score that discriminates between pathogenic and benign variants. All E555 variants were predicted to be pathogenic.

**References**

1. Murre C, McCaw PS, Baltimore D. A new DNA binding and dimerization motif in immunoglobulin enhancer binding, daughterless, MyoD, and myc proteins. Cell. 1989;56(5):777-83. https://doi.org/10.1016/0092-8674(89)90682-x
2. Sun XH, Baltimore D. An inhibitory domain of E12 transcription factor prevents DNA binding in E12 homodimers but not in E12 heterodimers. Cell. 1991;64(2):459-70. https://doi.org/10.1016/0092-8674(91)90653-g
3. Ephrussi A, Church GM, Tonegawa S, Gilbert W. B lineage-specific interactions of an immunoglobulin enhancer with cellular factors in vivo. Science. 1985;227(4683):134-40. https://doi.org/10.1126/science.3917574
4. Church GM, Ephrussi A, Gilbert W, Tonegawa S. Cell-type-specific contacts to immunoglobulin enhancers in nuclei. Nature. 1985;313(6005):798-801. https://doi.org/10.1038/313798a0
5. Murre C, McCaw PS, Vaessin H, Caudy M, Jan LY, Jan YN, et al. Interactions between heterologous helix-loop-helix proteins generate complexes that bind specifically to a common DNA sequence. Cell. 1989;58(3):537-44. https://doi.org/10.1016/0092-8674(89)90434-0
6. Lassar AB, Davis RL, Wright WE, Kadesch T, Murre C, Voronova A, et al. Functional activity of myogenic HLH proteins requires hetero-oligomerization with E12/E47-like proteins in vivo. Cell. 1991;66(2):305-15. https://doi.org/10.1016/0092-8674(91)90620-e
7. Blackwell TK, Weintraub H. Differences and similarities in DNA-binding preferences of MyoD and E2A protein complexes revealed by binding site selection. Science. 1990;250(4984):1104-10. https://doi.org/10.1126/science.2174572
8. Murre C, Bain G, van Dijk MA, Engel I, Furnari BA, Massari ME, et al. Structure and function of helix-loop-helix proteins. Biochim Biophys Acta. 1994;1218(2):129-35. https://doi.org/10.1016/0167-4781(94)90001-9
9. Lazorchak A, Jones ME, Zhuang Y. New insights into E-protein function in lymphocyte development. Trends Immunol. 2005;26(6):334-8. https://doi.org/10.1016/j.it.2005.03.011
10. Murre C, Voronova A, Baltimore D. B-cell- and myocyte-specific E2-box-binding factors contain E12/E47-like subunits. Mol Cell Biol. 1991;11(2):1156-60. https://doi.org/10.1128/mcb.11.2.1156-1160.1991
11. Shen CP, Kadesch T. B-cell-specific DNA binding by an E47 homodimer. Mol Cell Biol. 1995;15(8):4518-24. https://doi.org/10.1128/mcb.15.8.4518
12. Kee BL, Quong MW, Murre C. E2A proteins: essential regulators at multiple stages of B-cell development. Immunol Rev. 2000;175:138-49.
13. Bain G, Maandag EC, Izon DJ, Amsen D, Kruisbeek AM, Weintraub BC, et al. E2A proteins are required for proper B cell development and initiation of immunoglobulin gene rearrangements. Cell. 1994;79(5):885-92. https://doi.org/10.1016/0092-8674(94)90077-9
14. Zhuang Y, Cheng P, Weintraub H. B-lymphocyte development is regulated by the combined dosage of three basic helix-loop-helix genes, E2A, E2-2, and HEB. Mol Cell Biol. 1996;16(6):2898-905. https://doi.org/10.1128/mcb.16.6.2898
15. Greenbaum S, Zhuang Y. Regulation of early lymphocyte development by E2A family proteins. Semin Immunol. 2002;14(6):405-14. https://doi.org/10.1016/s1044532302000751
16. Murre C. Helix-loop-helix proteins and lymphocyte development. Nat Immunol. 2005;6(11):1079-86. https://doi.org/10.1038/ni1260
17. Beck K, Peak MM, Ota T, Nemazee D, Murre C. Distinct roles for E12 and E47 in B cell specification and the sequential rearrangement of immunoglobulin light chain loci. J Exp Med. 2009;206(10):2271-84. https://doi.org/10.1084/jem.20090756
18. Dobbs AK, Bosompem A, Coustan-Smith E, Tyerman G, Saulsbury FT, Conley ME. Agammaglobulinemia associated with BCR(-) B cells and enhanced expression of CD19. Blood. 2011;118(7):1828-37. https://doi.org/10.1182/blood-2011-01-330472
19. Boisson B, Wang YD, Bosompem A, Ma CS, Lim A, Kochetkov T, et al. A recurrent dominant negative E47 mutation causes agammaglobulinemia and BCR(-) B cells. J Clin Invest. 2013;123(11):4781-5. https://doi.org/10.1172/jci71927
20. Al Sheikh E, Arkwright PD, Herwadkar A, Hussell T, Briggs TA. TCF3 Dominant Negative Variant Causes an Early Block in B-Lymphopoiesis and Agammaglobulinemia. J Clin Immunol. 2021;41(6):1391-4. https://doi.org/10.1007/s10875-021-01049-9
21. Boast B, Goel S, Gonzalez-Granado LI, Niemela J, Stoddard J, Edwards ESJ, et al. TCF3 haploinsufficiency defined by immune, clinical, gene-dosage, and murine studies. J Allergy Clin Immunol. 2023;152(3):736-47. https://doi.org/10.1016/j.jaci.2023.05.017
22. Ben-Ali M, Yang J, Chan KW, Ben-Mustapha I, Mekki N, Benabdesselem C, et al. Homozygous transcription factor 3 gene (TCF3) mutation is associated with severe hypogammaglobulinemia and B-cell acute lymphoblastic leukemia. J Allergy Clin Immunol. 2017;140(4):1191-4 e4. https://doi.org/10.1016/j.jaci.2017.04.037
23. Qureshi S, Sheikh MDA, Qamar FN. Autosomal Recessive Agammaglobulinemia - first case with a novel TCF3 mutation from Pakistan. Clin Immunol. 2019;198:100-1. https://doi.org/10.1016/j.clim.2018.07.016
24. Khoshnevisan R, Hassanzadeh S, Klein C, Rohlfs M, Grimbacher B, Molavi N, et al. B-cells absence in patients diagnosed as inborn errors of immunity: a registry-based study. Immunogenetics. 2024;76(3):189-202. https://doi.org/10.1007/s00251-024-01342-y
25. Ameratunga R, Koopmans W, Woon ST, Leung E, Lehnert K, Slade CA, et al. Epistatic interactions between mutations of TACI (TNFRSF13B) and TCF3 result in a severe primary immunodeficiency disorder and systemic lupus erythematosus. Clin Transl Immunology. 2017;6(10):e159. https://doi.org/10.1038/cti.2017.41
26. Tangye SG, Al-Herz W, Bousfiha A, Cunningham-Rundles C, Franco JL, Holland SM, et al. Human Inborn Errors of Immunity: 2022 Update on the Classification from the International Union of Immunological Societies Expert Committee. J Clin Immunol. 2022;42(7):1473-507. https://doi.org/10.1007/s10875-022-01289-3
27. Picard C, Bobby Gaspar H, Al-Herz W, Bousfiha A, Casanova JL, Chatila T, et al. International Union of Immunological Societies: 2017 Primary Immunodeficiency Diseases Committee Report on Inborn Errors of Immunity. J Clin Immunol. 2018;38(1):96-128. https://doi.org/10.1007/s10875-017-0464-9
28. Bousfiha A, Jeddane L, Picard C, Ailal F, Bobby Gaspar H, Al-Herz W, et al. The 2017 IUIS Phenotypic Classification for Primary Immunodeficiencies. J Clin Immunol. 2018;38(1):129-43. https://doi.org/10.1007/s10875-017-0465-8
29. El Omari K, Hoosdally SJ, Tuladhar K, Karia D, Hall-Ponsele E, Platonova O, et al. Structural basis for LMO2-driven recruitment of the SCL:E47bHLH heterodimer to hematopoietic-specific transcriptional targets. Cell Rep. 2013;4(1):135-47. https://doi.org/10.1016/j.celrep.2013.06.008
30. Takashima T, Okamura M, Yeh TW, Okano T, Yamashita M, Tanaka K, et al. Multicolor Flow Cytometry for the Diagnosis of Primary Immunodeficiency Diseases. J Clin Immunol. 2017;37(5):486-95. https://doi.org/10.1007/s10875-017-0405-7
31. Tadaka S, Kawashima J, Hishinuma E, Saito S, Okamura Y, Otsuki A, et al. jMorp: Japanese Multi-Omics Reference Panel update report 2023. Nucleic Acids Res. 2024;52(D1):D622-D32. https://doi.org/10.1093/nar/gkad978
32. Cunningham BC, Wells JA. High-resolution epitope mapping of hGH-receptor interactions by alanine-scanning mutagenesis. Science. 1989;244(4908):1081-5. https://doi.org/10.1126/science.2471267
33. Kagawa R, Fujiki R, Tsumura M, Sakata S, Nishimura S, Itan Y, et al. Alanine-scanning mutagenesis of human signal transducer and activator of transcription 1 to estimate loss- or gain-of-function variants. J Allergy Clin Immunol. 2017;140(1):232-41. https://doi.org/10.1016/j.jaci.2016.09.035
34. Voronova A, Baltimore D. Mutations that disrupt DNA binding and dimer formation in the E47 helix-loop-helix protein map to distinct domains. Proc Natl Acad Sci U S A. 1990;87(12):4722-6. https://doi.org/10.1073/pnas.87.12.4722
35. Ellenberger T, Fass D, Arnaud M, Harrison SC. Crystal structure of transcription factor E47: E-box recognition by a basic region helix-loop-helix dimer. Genes Dev. 1994;8(8):970-80. https://doi.org/10.1101/gad.8.8.970
36. Maerkl SJ, Quake SR. Experimental determination of the evolvability of a transcription factor. Proc Natl Acad Sci U S A. 2009;106(44):18650-5. https://doi.org/10.1073/pnas.0907688106
37. De Masi F, Grove CA, Vedenko A, Alibes A, Gisselbrecht SS, Serrano L, et al. Using a structural and logics systems approach to infer bHLH-DNA binding specificity determinants. Nucleic Acids Res. 2011;39(11):4553-63. https://doi.org/10.1093/nar/gkr070
38. O'Shea EK, Rutkowski R, Kim PS. Mechanism of specificity in the Fos-Jun oncoprotein heterodimer. Cell. 1992;68(4):699-708. https://doi.org/10.1016/0092-8674(92)90145-3
39. O'Shea EK, Lumb KJ, Kim PS. Peptide 'Velcro': design of a heterodimeric coiled coil. Curr Biol. 1993;3(10):658-67. https://doi.org/10.1016/0960-9822(93)90063-t
40. Nohaile MJ, Hendsch ZS, Tidor B, Sauer RT. Altering dimerization specificity by changes in surface electrostatics. Proc Natl Acad Sci U S A. 2001;98(6):3109-14. https://doi.org/10.1073/pnas.051624498
41. Escherich C, Chen W, Miyamoto S, Namikawa Y, Yang W, Teachey DT, et al. Identification of TCF3 germline variants in pediatric B-cell acute lymphoblastic leukemia. Blood Adv. 2023;7(10):2177-80. https://doi.org/10.1182/bloodadvances.2022008563
42. Rapaport F, Boisson B, Gregor A, Beziat V, Boisson-Dupuis S, Bustamante J, et al. Negative selection on human genes underlying inborn errors depends on disease outcome and both the mode and mechanism of inheritance. Proc Natl Acad Sci U S A. 2021;118(3). https://doi.org/10.1073/pnas.2001248118
43. Gussow AB, Petrovski S, Wang Q, Allen AS, Goldstein DB. The intolerance to functional genetic variation of protein domains predicts the localization of pathogenic mutations within genes. Genome Biol. 2016;17:9. https://doi.org/10.1186/s13059-016-0869-4
44. Brandes N, Goldman G, Wang CH, Ye CJ, Ntranos V. Genome-wide prediction of disease variant effects with a deep protein language model. Nat Genet. 2023;55(9):1512-22. https://doi.org/10.1038/s41588-023-01465-0
